# Identification of IRF1 as critical dual regulator of Smac mimetic-induced apoptosis and inflammatory cytokine response

**DOI:** 10.1038/cddis.2014.498

**Published:** 2014-12-11

**Authors:** I Eckhardt, A Weigert, S Fulda

**Affiliations:** 1 Institute for Experimental Cancer Research in Pediatrics, Goethe-University, Komturstrasse 3a, 60528 Frankfurt, Germany; 2 Institute of Biochemistry I, Goethe-University, Sandhofstraße, 60528 Frankfurt, Germany

## Abstract

Smac (second mitochondria-derived activator of caspase) mimetics are considered as promising anticancer therapeutics and used to induce apoptosis by antagonizing inhibitor of apoptosis proteins, which are often abundantly expressed in cancer cells. Here, we identify interferon regulatory factor 1 (IRF1) as a novel critical regulator of Smac mimetic BV6-induced apoptosis and proinflammatory cytokine secretion with impact on the immune response. IRF1 knockdown rescues cells from BV6-induced apoptosis and attenuates BV6-stimulated upregulation of tumor necrosis factor-*α* (TNF*α*), indicating that IRF1 mediates BV6-triggered cell death, at least in part, by inducing TNF*α*. This notion is supported by data showing that exogenous supply of TNF*α* restores BV6-induced cell death in IRF-knockdown cells. Interestingly, IRF1 selectively controls the induction of nuclear factor-*κ*B (NF-*κ*B) target genes, as IRF1 depletion attenuates BV6-stimulated upregulation of TNF*α* and interleukin-8 (IL-8) but not p100 and RelB. Concomitant knockdown of IRF1 and p65 cooperate to inhibit BV6-induced cell death, implying a cooperative interaction of IRF1 and NF-*κ*B. In addition, IRF1 silencing hampers TNF*α* induction by TNF*α* itself as an another prototypical NF-*κ*B stimulus. Importantly, IRF1 depletion impedes BV6-stimulated secretion of additional proinflammatory cytokines such as granulocyte–macrophage colony-stimulating factor (GM-CSF), IL-8, IL-6 and monocyte chemoattractant protein-1, and migration of primary monocytes to BV6-treated tumor cells. In conclusion, this identification of IRF1 as a dual regulator of BV6-induced apoptosis and inflammatory cytokine secretion provides novel insights into determinants of sensitivity towards Smac mimetic and possible implications of Smac mimetic treatment on tumor microenvironment and immune response.

Apoptosis is a critical mechanism for cellular homeostasis, and evasion from apoptosis is one hallmark of human cancers.^1^ Cancer cells prevent cell death induction by deregulation of multiple components of the apoptosis signaling network such as inhibitor of apoptosis (IAP) proteins.^2^

IAP proteins are often abundantly expressed in various cancers.^2^ They are characterized by harboring at least one baculovirus IAP repeat domain (BIR) and by inhibiting apoptosis in multiple ways.^2^ X-linked IAP protein (XIAP) blocks caspase activity via direct binding, whereas cellular IAP (cIAP) proteins possess E3-ligase activity because of their RING (really interesting new gene) domain and regulate canonical and noncanonical nuclear factor-*κ*B (NF-*κ*B) signaling by ubiquitination.^2^ cIAP-mediated K63-linked polyubiquitination regulates receptor-interacting protein 1 (RIP1) function and is necessary for tumor necrosis factor receptor 1 (TNFR1)-stimulated activation of the canonical NF-*κ*B pathway.^3, 4^ Phosphorylation and degradation of I*κ*B*α* releases p50/p65 dimers from inhibition, and favors their nuclear translocation and transactivation of NF-*κ*B target genes.^4^ On the contrary, cIAP proteins inhibit noncanonical NF-*κ*B activation by constitutively ubiquitinating NF-*κ*B-inducing kinase (NIK) in a complex together with TNFR-associated factor 2 (TRAF2) and TRAF3, thereby marking it for proteasomal degradation.^5^

Upon apoptosis induction, IAP proteins are antagonized by their endogenous inhibitor Smac (second mitochondria-derived activator of caspase), which is released from the mitochondrial intermembrane space, enabling the activation of caspases.^2^ The development of pharmacological IAP inhibitors such as Smac mimetics is considered as a promising therapeutic approach to promote apoptosis in cancer cells.^2^ Smac mimetics neutralize the inhibitory function of XIAP and favor autoubiquitination and subsequent degradation of cIAP1/2.^6, 7^ In addition to activation of caspases, Smac mimetics have been described to trigger an NF-*κ*B-dependent autocrine/paracrine TNF*α* loop, which induces cell death by facilitating the formation of a TNF*α*-induced cytosolic complex II consisting of caspase-8, Fas-associated protein with death domain and RIP1, which drives caspase-8 activation and cell death.^6, 7^

The interferon regulatory factor (IRF) family is a class of transcription factors involved in the regulation of immune processes and oncogenesis.^8^ To date, there are nine family members that all contain a helix–turn–helix N-terminal DNA-binding domain and a regulatory C-terminal portion, which mediates interaction with each other and other transcription factors.^8^ IRFs bind to interferon-stimulated response elements and have been implicated in the transactivation or repression of target genes.

IRF1 was the first member identified by its transcriptional regulation of the interferon-*β* (*IFNβ*) gene^9^ and is one of the best characterized family members. IRF1 is a short-lived protein with a half-life of ~30 min owing to the K48-linked polyubiquitination at its C terminus and subsequent proteasomal degradation.^10^ IRF1 can be transcriptionally upregulated upon activation of the JAK/STAT or NF-*κ*B pathway^11, 12, 13, 14^ or in response to genotoxic stress.^15^ Besides its well-described function in the regulation of IFN-inducible genes, IRF1 has been reported to act as a tumor suppressor that regulates the expression of genes involved in apoptosis, cell growth control and angiogenesis.^16, 17, 18, 19^ Furthermore, interaction of IRF1 with other transcription factors, including NF-*κ*B subunits p50 and p65, has been shown to modulate target and stimulus specificity.^20, 21^

To exploit Smac mimetics as anticancer therapeutics, it is pivotal to understand their molecular mechanism of action. Therefore, we aimed to identify novel key regulators of Smac mimetic-induced apoptosis in the present study.

## Results

### IRF1 is necessary for BV6-mediated cell death

To gain new insights into the molecular mechanisms underlying BV6-induced cell death, we performed whole-genome expression profiling in an NF-*κ*B-proficient and -deficient system to filter BV6-inducible, NF-*κ*B-regulated factors, as we previously described a proapoptotic function of NF-*κ*B in Smac mimetic-induced cell death.^22, 23, 24^ Using this approach, we identified IRF1 as one of the top 10 upregulated NF-*κ*B target genes upon treatment with BV6 (Supplementary Figure 1 and Supplementary Table 2). As IRF1 has been implicated as an inducer of apoptosis,^16, 18, 25^ we decided to explore the role of IRF1 in the context of BV6-triggered apoptosis in more detail.

**Figure 1:**
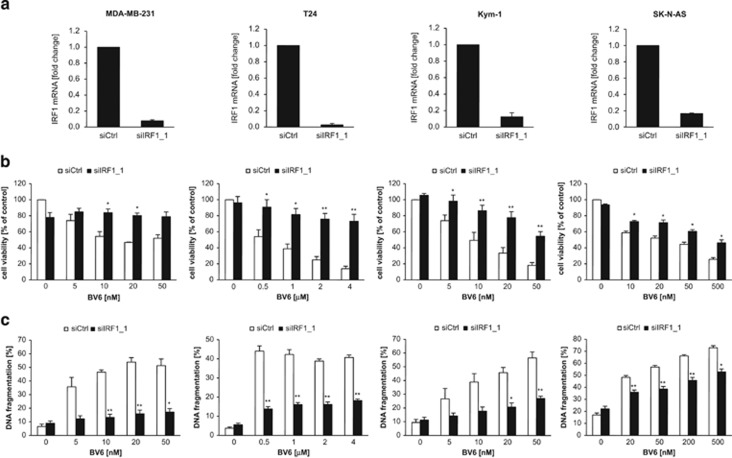
IRF1 is necessary for BV6-mediated cell death. MDA-MB-231, T24, Kym1 and SK-N-AS cells were transiently transfected with 5 nM siRNA against IRF1 or control siRNA. (**a**) IRF1 mRNA levels were analyzed after 24 h by quantitative reverse transcriptase-PCR (qRT-PCR) and normalized to 28S rRNA expression. Data are presented as fold change of control siRNA. Mean±S.D. of at least two independent experiments performed in duplicate are shown. (**b** and **c**) Cells were treated with indicated concentrations of BV6 for 72 h. Cell viability was measured by MTT and is expressed as the percentage of untreated controls (**b**) Apoptosis was determined by DNA fragmentation of PI-stained nuclei using flow cytometry and the percentage of DNA fragmentation with mean±S.E.M. of three to five independent experiments performed in duplicate is shown (**c**). *P<0.05; **P<0.01

To test whether IRF1 is required for BV6-induced cell death, we monitored different parameters of cell death in a panel of IRF1-depleted cancer cell lines (Figure 1a). Control experiments confirmed the specificity of IRF1 siRNA to silence IRF1 compared with other IRF family members (Supplementary Figure 2). Importantly, IRF1 knockdown significantly rescued cells from BV6-induced loss of cell viability and apoptosis (Figures 1b and c and Supplementary Figures 3 and 4A). Induction of apoptosis was confirmed by Annexin-V/propidium iodide (PI) staining (Supplementary Figures 4B and 5). These findings support our hypothesis based on our gene expression profiling data that IRF1 represents a critical regulator of BV6-triggered cell death.

### IRF1 is indispensable for BV6-mediated TNF*α* induction

Next, we aimed to identify the mechanism underlying the requirement of IRF1 for BV6-mediated cell death. As TNF*α* production has been shown to be necessary for Smac mimetic-induced cell death,^6, 7^ we tested whether TNF*α* is required for BV6-induced cell death in our cell systems. Treatment with BV6 stimulated the production of TNF*α* mRNA as well as the secretion of TNF*α* protein (Supplementary Figure 6). TNFR1 silencing using two independent siRNA sequences efficiently protected cells from BV6-induced loss of cell viability and apoptosis^26^ (Supplementary Figure 7). Consistently, we previously demonstrated that the addition of the TNF*α*-blocking antibody Enbrel protected against BV6-induced cell death.^26^ These data confirm that TNF*α* is required for BV6-mediated cell death in our cell lines.

**Figure 2:**
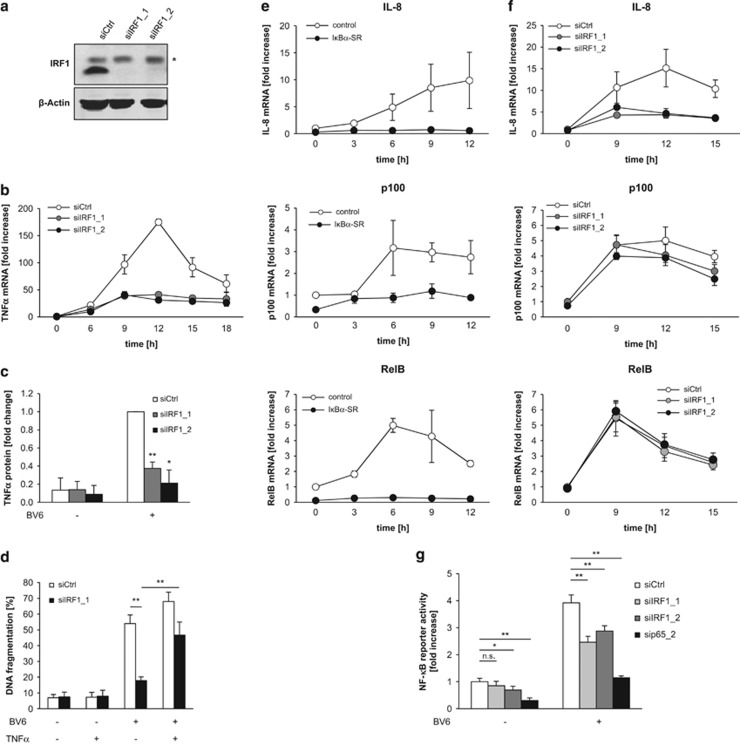
IRF1 is indispensable for BV6-mediated TNF*α* induction and selectively controls BV6-induced NF-*κ*B target genes. MDA-MB-231 cells were transiently transfected with 5 nM siRNA against IRF1 or control siRNA. (**a**) Protein levels of IRF1 were analyzed after 24 h by western blotting. *β*-Actin served as a loading control. (**b**) Cells were treated with 50 nM BV6 for indicated time points. TNF*α* mRNA levels were assessed by quantitative reverse transcriptase-PCR (qRT-PCR) and normalized to 28S rRNA expression (**b**). Data are presented as fold increase of untreated control siRNA cells. Mean±S.E.M. of two independent experiments performed in duplicate are shown. (**c**) Cells were treated with 50 nM BV6 for 15 h, cell culture supernatants were harvested and assessed for TNF*α* protein levels by ELISA. Data are presented as fold change of treated control siRNA cells. Mean±S.D. of three independent experiments performed in duplicate are shown. (**d**) Cells were treated with 50 nM BV6 for 15 h. TNF*α* of 50 pg/ml was additionally added after 15 h and cells were analyzed after 48 h in total. Apoptosis was determined by DNA fragmentation of PI-stained nuclei using flow cytometry and the percentage of DNA fragmentation is shown with mean±S.D. of three independent experiments performed in duplicate. (**e**) MDA-MB-231 cells stably expressing I*κ*B*α*-SR or vector control were treated for indicated time points with 50 nM BV6. IL-8, p100 and RelB mRNA levels were assessed by qRT-PCR and normalized to 28S rRNA expression. Data are presented as fold increase of untreated vector control cells. Mean±S.E.M. of two independent experiments performed in duplicate are shown. (**f**) MDA-MB-231 cells were transiently transfected with 5 nM siRNA against IRF1 or control siRNA and were treated for indicated time points with 50 nM BV6. IL-8, p100 and RelB mRNA levels were assessed by qRT-PCR and normalized to 28S rRNA expression. Data are presented as fold increase of untreated control siRNA cells. Mean±S.E.M. of three independent experiments performed in duplicate are shown. (**g**) MDA-MB-231 cells stably expressing a GFP-labeled NF-*κ*B reporter construct were transiently transfected with 5 nM siRNA against IRF1, p65 or control siRNA and treated for 15 h with 50 nM BV6. NF-*κ*B transcriptional activity was assessed by flow cytometry and is displayed as fold increase of untreated control siRNA cells. Mean±S.D. of three independent experiments performed in duplicate are shown. *P<0.05; **P<0.01

Next, we explored whether IRF1 is required for BV6-mediated upregulation of TNF*α*. Importantly, IRF1 knockdown prevented the maximal induction of TNF*α* mRNA levels upon BV6 treatment (Figures 2a and b and Supplementary Figures 8A and B). In addition, IRF1 knockdown significantly reduced the BV6-stimulated secretion of TNF*α* into the cell culture supernatant (Figure 2c and Supplementary Figure 8C), confirming that changes in TNF*α* mRNA levels translate to protein expression. These experiments show that IRF1 is indispensable for BV6-induced upregulation of TNF*α* mRNA and protein expression.

To investigate whether IRF1-stimulated TNF*α* expression is responsible for BV6-induced apoptosis, we tested whether the supply of exogenous TNF*α* restores BV6-mediated cell death in IRF1-knockdown cells based on our findings that these cells are defective in TNF*α* upregulation upon treatment with BV6 (Figures 2b and c and Supplementary Figures 8B and C). To mimic the endogenous TNF*α* response upon BV6 treatment, which stimulates the autocrine/paracrine production of TNF*α* in BV6-sensitive tumor cells^26^ (Supplementary Figure 7), we applied low doses of TNF*α* with a time delay of 15 h after BV6 treatment. Intriguingly, addition of TNF*α* reversed the protection provided by IRF1 silencing and substantially restored BV6-mediated cell death in IRF1-depleted cells (Figure 2d and Supplementary Figure 8D). Taken together, these experiments indicate that IRF1 mediates BV6-induced cell death by upregulating TNF*α*.

### IRF1 selectively controls BV6-induced NF-*κ*B target genes

As we previously reported that BV6 induces various NF-*κ*B-regulated target genes,^27, 28^ we wondered to which extent IRF1 is required for this BV6-stimulated NF-*κ*B response. For this purpose, we monitored the expression levels of three typical NF-*κ*B target genes upon BV6 treatment. BV6 triggered the induction of interleukin-8 (IL-8), p100 and RelB in an NF-*κ*B-dependent manner, as inhibition of NF-*κ*B by overexpression of I*κ*B*α*-superrepressor (SR) overexpression abrogated this upregulation (Figure 2e). Interestingly, knockdown of IRF1 only efficiently prevented BV6-mediated IL-8 induction, whereas upregulation of p100 and RelB remained largely unaffected (Figure 2f and Supplementary Figure 9). Consistently, IRF1 silencing partially reduced BV6-stimulated NF-*κ*B transcriptional activity, whereas knockdown of p65, as key component of NF-*κ*B signaling, almost completely abolished both basal and BV6-induced NF-*κ*B transcriptional activity (Figure 2g). These findings support the notion that IRF1 selectively regulates a distinct set of BV6-induced NF-*κ*B target genes and contributes to NF-*κ*B activation.

### IRF1 cooperates with p65 in BV6-induced cell death

**Figure 3:**
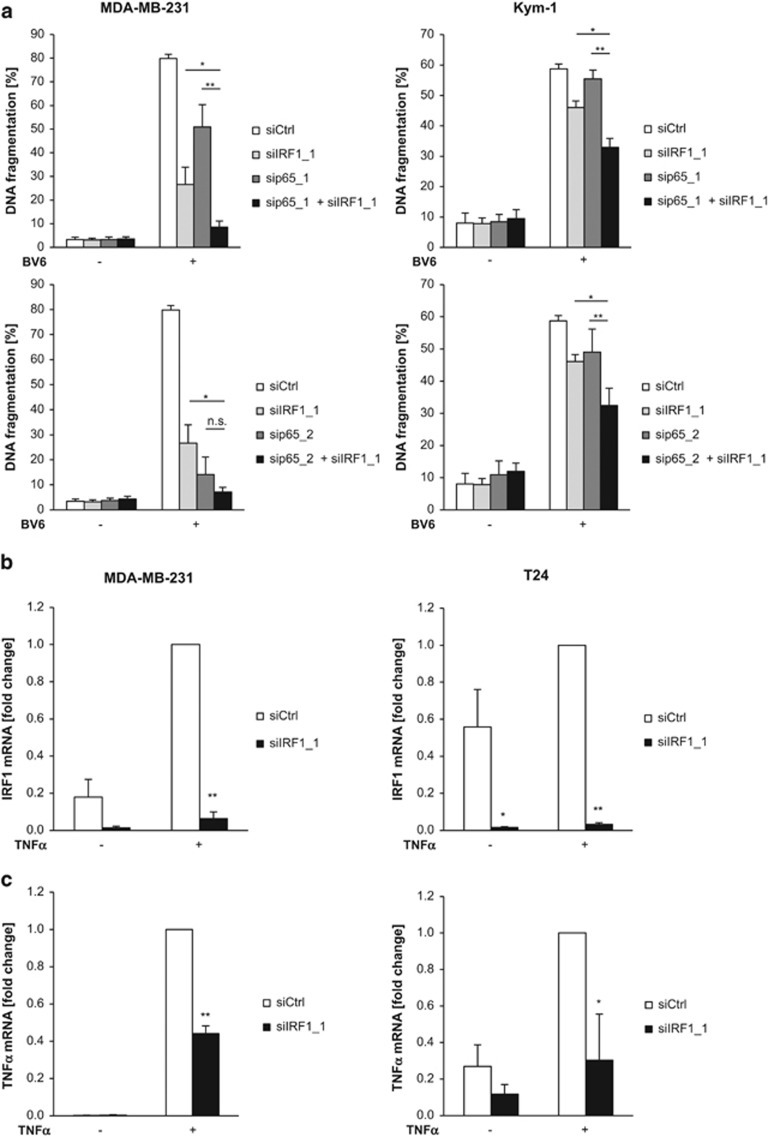
IRF1 cooperates with NF-*κ*B in BV6-induced cell death. (**a**) MDA-MB-231 and Kym1 cells were transiently transfected with 5 nM siRNA against IRF1, p65 or control siRNA and treated with 50 nM BV6 (MDA-MB-231) or 100 nM BV6 (Kym1) for 72 h. Apoptosis was determined by DNA fragmentation of PI-stained nuclei using flow cytometry and the percentage of DNA fragmentation is shown with mean±S.D. of three independent experiments performed in duplicate. (**b** and **c**) MDA-MB-231 and T24 cells were transiently transfected with 5 nM siRNA against IRF1 or control siRNA and treated with 1 ng/ml TNF*α* for 2 h. mRNA levels of IRF1 (**b**) and TNF*α* (**c**) were assessed by quantitative reverse transcriptase-PCR qRT-PCR and normalized to 28S rRNA expression. Data are presented as fold change of treated control siRNA cells. Mean±S.D. of three to four independent experiments performed in duplicate are shown. *P<0.05; **P<0.01

To address the question whether IRF1 and NF-*κ*B cooperate in BV6-induced cell death, we concomitantly knocked down IRF1 and p65 (Supplementary Figure 10). Importantly, simultaneous knockdown of IRF1 and p65 cooperated to decrease BV6-mediated cell death (Figure 3a). Consistently, double knockdown most efficiently hampered BV6-stimulated induction of TNF*α* mRNA levels and NF-*κ*B transcriptional activity (Supplementary Figure 11). These data confirm a cooperative role of IRF1 and p65 in BV6-induced cell death. By comparison, silencing of other NF-*κ*B subunits such as RelB or c-Rel had no or moderate effects on BV6-induced cell death (Supplementary Figure 12).

### IRF1 is required for TNF*α*-mediated TNF*α* induction

We next asked whether IRF1 is also required for the induction of TNF*α* in response to other stimuli that activate NF-*κ*B. As TNF*α* is a prototypical NF-*κ*B stimulus that engages a self-sustaining feedforward cycle, we used TNF*α* to test this hypothesis. To this end, we compared TNF*α*-stimulated production of TNF*α* in control and IRF1-depleted cells. Of note, knockdown of IRF1 significantly attenuated TNF*α*-stimulated induction of TNF*α* mRNA (Figures 3b and c). This experiment showing that TNF*α* induction by a different NF-*κ*B stimulus also depends on IRF1 suggests that IRF1 more generally regulates TNF*α* expression.

### Minor contribution of IRF5 to BV6-induced cell death

Next, we also explored whether other IRF family members are involved in BV6-induced cell death and cytokine production. IRF5 has recently been implicated in the sustained TNF*α* response in dendritic cells upon lipopolysaccharide (LPS) stimulation.^29^ To explore whether IRF5 contributes to BV6-induced cell death, we knocked down IRF5 by two independent siRNA sequences (Supplementary Figure 13A). Depletion of IRF5 somewhat reduced BV6-triggered cell death as well as the upregulation of TNF*α* (Supplementary Figures 13B and C). By comparison, BV6 did not substantially alter IRF5 mRNA levels (Supplementary Figure 13D). These findings demonstrate that IRF5 has a minor contribution to BV6-stimulated cell death and TNF*α* expression.

### BV6 induces nuclear accumulation of IRF1

Next, we investigated how BV6 treatment regulates IRF1 expression and activity. As the *IRF1* gene is located on 5q31.1, a region that frequently displays copy number changes in tumors,^30, 31, 32, 33^ we examined whether the cell lines used in this study harbor a genetic alteration at the IRF1 locus. Analysis of IRF1 locus copy number revealed the same physiological number of gene copies in MDA-MB-231 and T24 cells compared with non-malignant HEK293T cells (Supplementary Figure 14), demonstrating that there is no genetic alteration at the IRF1 locus in these tumor cell lines.

Next, we explored whether BV6 treatment triggers upregulation of IRF1 mRNA levels. BV6 treatment significantly increased IRF1 mRNA levels in MDA-MB-231 and SK-N-AS cells (Supplementary Figure 15A). By comparison, IRF1 mRNA levels were slightly, but not significantly increased by BV6 in T24 cells (Supplementary Figure 15A), pointing to a context-dependent regulation of IRF1 mRNA levels by BV6.

As IRF1 is known as a short-lived protein with high proteasomal turnover,^10^ we asked whether BV6 affects the half-life of IRF1. To address this question, we performed cycloheximide (CHX) chase experiments. Cells were pretreated with BV6 for 3 h before CHX was applied to prevent *de novo* protein synthesis of IRF1. Using this approach, we did not detect alterations in protein turnover of IRF1 upon BV6 treatment (Supplementary Figure 15B).

To explore whether BV6 affects the nuclear localization of IRF1, we prepared nuclear and cytosolic cell extracts. Treatment with BV6 resulted in a slight increase of IRF1 protein in the nucleus (Supplementary Figures 15C and 5D). We also noted that IRF1 was constitutively expressed exclusively in the nucleus (Supplementary Figure 15C).

Taken together, this set of experiments suggests that BV6 slightly increases nuclear localization of IRF1, whereas upregulation of IRF1 mRNA levels by BV6 depends on the context.

### IRF1 is required for BV6-induced secretion of proinflammatory cytokines and recruitment of monocytes

**Figure 4:**
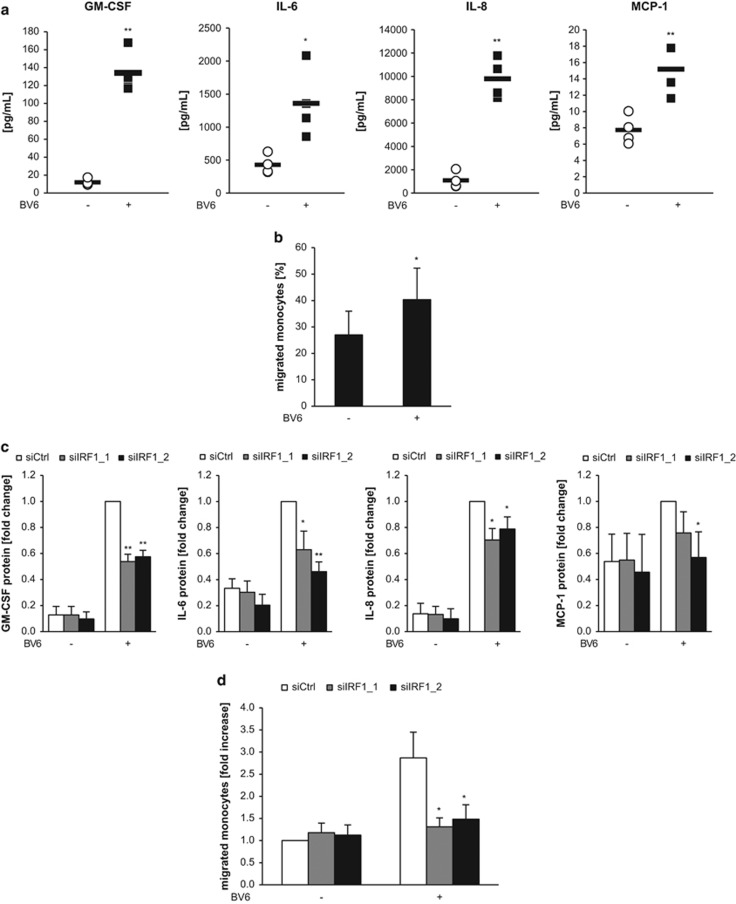
IRF1 is required for BV6-induced secretion of proinflammatory cytokines and recruitment of monocytes. (**a**) MDA-MB-231 cells were treated with 50 nM BV6 supplemented with 20 *μ*M zVAD.fmk for 15 h and cell culture supernatants were harvested. GM-CSF, IL-6, IL-8 and MCP-1 levels were assessed by CBA and flow cytometry. Mean±S.D. of four independent experiments are shown. (**b**) MDA-MB-231 cells were treated with 50 nM BV6 for 3 h. Medium was replaced by fresh drug-free and reduced serum (0.1%) medium supplemented with 20 *μ*M zVAD.fmk and incubated overnight. A total of 10^6^ freshly isolated PBMCs were applied in transwells and allowed to migrate for 3 h. Percentage of migrated monocytes was assessed by flow cytometry. Mean±S.D. values of PBMCs from four different donors are shown. (**c**) MDA-MB-231 cells were transiently transfected with 5 nM siRNA against IRF1 or control siRNA, treated with 50 nM BV6 supplemented with 20 *μ*M zVAD.fmk for 15 h and cell culture supernatants were harvested. GM-CSF, IL-6, IL-8 and MCP-1 levels were assessed by CBA and flow cytometry. Data are presented as fold change of treated control siRNA cells. Mean±S.D. of four independent experiments are shown. (**d**) MDA-MB-231 cells were transiently transfected with 5 nM siRNA against IRF1 or control siRNA and treated with 50 nM BV6 for 3 h. Medium was replaced by fresh drug-free and reduced serum (0.1%) medium supplemented with 20 *μ*M zVAD.fmk and incubated overnight. A total of 10^6^ freshly isolated PBMCs were applied in transwells and allowed to migrate for 3 h. Percentage of migrated monocytes was assessed by flow cytometry. Data are presented as fold increase of untreated control siRNA cells. Mean±S.E.M. of two transfections with PBMCs from two to three different donors each are shown. *P<0.05; **P<0.01

As both NF-*κ*B and IRFs are involved in the regulation of immune processes, we investigated the effects of BV6 treatment on the induction of additional proinflammatory cytokines and possible implications on the immune system. Importantly, BV6 significantly increased the secretion of IL-8, IL-6, granulocyte–macrophage colony-stimulating factor (GM-CSF) and monocyte chemoattractant protein-1 (MCP-1) from tumor cells into the supernatant (Figure 4a), whereas IL-10 and VEGF remained largely unchanged (data not shown).

As these cytokines have been implicated in the recruitment of innate immune cells to the tumor site, we asked whether treatment of tumor cells with BV6 influences the attraction of monocytes. To address this question, we treated MDA-MB-231 cells with BV6 for 3 h to stimulate cytokine production followed by replacement with fresh, drug-free and reduced serum (0.1%) medium to avoid possible effects of BV6 and serum factors on immune cells. After overnight incubation, we applied peripheral blood mononucleated cells (PBMCs) from healthy donors in transwell inserts, which allow the diffusion of soluble factors, and let them migrate towards tumor cells. Interestingly, BV6 pretreatment of tumor cells significantly augmented the percentage of monocytes migrating towards tumor cells (Figure 4b). This suggests that BV6-induced cytokine secretion by tumor cells into the supernatant promotes the recruitment of monocytes.

Finally, we investigated whether IRF1 is required for BV6-mediated cytokine induction and monocyte recruitment. Interestingly, IRF1 depletion in tumor cells significantly attenuated BV6-stimulated secretion of IL-8, IL-6, GM-CSF and MCP-1 by tumor cells (Figure 4c). Importantly, IRF1 knockdown in tumor cells significantly impeded migration of monocytes towards BV6-treated tumor cells (Figure 4d). This set of experiments indicates that IRF1 expression in tumor cells is required for BV6-induced cytokine secretion by tumor cells and recruitment of monocytes towards tumor cells.

## Discussion

To exploit Smac mimetics as anticancer therapeutics, it is pivotal to understand their molecular mechanism of action. The novelty of the present study resides in the identification of IRF1 as a key dual regulator of BV6-mediated cell death and proinflammatory cytokine secretion with impact on the immune response using genome-wide gene expression profiling.

Several lines of evidence support this conclusion. First, IRF1 expression is indispensable for BV6-induced cell death in a panel of cancer cell lines, as siRNA-mediated depletion of IRF1 significantly rescues cells from BV6-induced loss of cell viability and apoptosis. Second, IRF1 mediates BV6-induced cell death, at least in part, by upregulating TNF*α*, as IRF1 knockdown attenuates BV6-stimulated TNF*α* upregulation in cells, which die in a TNF*α*-dependent manner upon treatment with BV6. In addition, exogenous supply of TNF*α* restores BV6-induced cell death in IRF1-knockdown cells. Third, IRF1 differentially regulates the BV6-stimulated NF-*κ*B response and is necessary for full NF-*κ*B transcriptional activity, as IRF1 silencing selectively prevents BV6-mediated induction of NF-*κ*B target genes, such as *TNFα* and *IL-8*, and attenuates BV6-stimulated NF-*κ*B transcriptional activation. Fourth, IRF1 is required for the BV6-stimulated secretion of inflammatory cytokines and immune response, as IRF1 silencing in tumor cells attenuates cytokine secretion by BV6-treated cells as well as by migration of primary monocytes towards tumor cells. Taken together, these data demonstrate that IRF1 has a dual role in BV6-mediated signaling: It acts as a proapoptotic factor in cell death induction and also affects the interaction of tumor cells with their microenvironment by promoting the secretion of cytokines and attraction of immune cells.

Our study reveals that IRF1 serves as a transcriptional activator, besides NF-*κ*B, that is essential to fully engage the BV6-triggered apoptotic program. Although NF-*κ*B has so far been implied in activating an autocrine/paracrine TNF*α* loop that drives Smac mimetic-induced apoptosis,^6, 7^ we now identify IRF1 as an additional transcriptional activator that is activated by BV6 and indispensable for full induction of TNF*α* and apoptosis. This conclusion is supported by our data showing that (1) IRF1 knockdown inhibits BV6-stimulated upregulation of TNF*α* and apoptosis, and that (2) the supply of exogenous TNF*α* restores BV6-mediated apoptosis in IRF1-depleted cells. IRF family members have previously been implicated in transcriptional regulation of TNF*α*. For example, IFNγ-triggered TNF*α* induction in mouse macrophages has been attributed to a cooperative regulation by IRF1 and IRF8^34^ and IRF5 has been linked to LPS-induced TNF*α* upregulation in dendritic cells.^29^ Additionally, IRF3 and TRIF (Toll/IL-1 receptor domain-containing adaptor-inducing IFN*β*) were reported to activate the TNF*α* promoter in a chronic ethanol abuse model.^35^

Moreover, our study demonstrates that IRF1 expression in tumor cells controls BV6-stimulated secretion of several proinflammatory cytokines by tumor cells, which alters their communication with components of the immune system by triggering the recruitment of monocytes. IRF1 is required for this interaction of tumor cells with the microenvironment, as IRF1 depletion abolishes BV6-stimulated cytokine secretion and monocyte attraction. Genetic or pharmacological inhibition of IAP proteins has previously been shown to regulate spontaneous and TNF*α*-stimulated cytokine and chemokine production.^36^ Furthermore, IRF1 expression in tumor cells was reported to mediate the crosstalk between tumor cells and natural killer (NK) cells by promoting the attraction of NK cells via increased secretion of the chemokine CXCL11, thereby contributing to immunosurveillance in the metastatic niche.^37^ Ectopic expression of a cytosolic form of Smac has been described to promote tumor immunity by eliciting a proinflammatory cell death in cancer cells that engages an adaptive antitumor immune response via exposure of calreticulin.^38^ Thus, Smac may modulate tumor immunity via its cytotoxic as well as its non-apoptotic effects on tumor cells. The observed non-apoptotic role of IRF1 in BV6-stimulated cytokine secretion in the present study is in line with our recent reports showing that Smac mimetic at non-toxic concentrations increases migration and invasion of glioblastoma cells in an autocrine/paracrine manner^27^ and promotes glioblastoma cancer stem-like cell differentiation.^28^

IRF1 is known as an NF-*κ*B target gene and has been reported to be upregulated by various NF-*κ*B stimuli such as CD40^11^ or TNF*α* in combination with prostaglandin E2.^39^ Our data showing that BV6 induces upregulation of IRF1 mRNA in a context-dependent manner in the investigated cell lines, while it slightly increases IRF1 protein expression in the nucleus, are consistent with additional, posttranscriptional mechanisms of IRF1 regulation. DNA binding of IRF1 has previously been reported to prevent its ubiquitination and protects the DNA-bound pool of IRF1 from proteasomal degradation.^40^ However, BV6 treatment had no detectable effects on the half-life of IRF1, which is known as an unstable protein because of its constitutive K48-linked polyubiquitination and subsequent proteasome-dependent degradation.^10^ Thus, additional studies are required to understand the context-dependent mechanisms of IRF1 regulation by BV6.

Components of the IFN signaling network have been described to directly interact with and to cooperatively induce target genes together with subunits of the NF-*κ*B signaling network.^20, 21, 41^ Our study shows that IRF1 contributes to and modulates BV6-mediated NF-*κ*B activation, as IRF1 silencing selectively attenuates BV6-stimulated upregulation of *bona fide* NF-*κ*B target genes such as *TNFα* and *IL-8*, and dampens the NF-*κ*B transcriptional activity. Furthermore, our data demonstrate a functional cooperativity of IRF1 and p65, as concomitant knockdown of both components significantly reduces BV6-stimulated NF-*κ*B transcriptional activity, TNF*α* induction and cell death. Thus, several transcription factors including NF-*κ*B and IRF1 control the induction of TNF*α* and cell death by Smac mimetics.

By identifying IRF1 as a key mediator of BV6-induced cell death and cytokine response, our study contributes to an improved understanding of factors that determine sensitivity to Smac mimetics and predicts implications on the tumor microenvironment and immune system. The requirement of IRF1 for both BV6-initiated apoptosis and immune response might be ambivalent as it activates BV6-induced cell death, but it also favors a proinflammatory response with local and potentially also systemic consequences. On the one hand, IRF1 expression in tumor cells has been demonstrated to be essential for tumor immunosurveillance by promoting NK cell attraction via chemokine secretion by tumor cells.^37^ On the other hand, cytokines including TNF*α*, IL-6 and IL-8 have been implicated in tumor progression via cancer-association inflammation, which involved the recruitment of immune cells to the tumor site where they were shown to support tumor growth.^42^ Further studies are required to determine the implications of the BV6-stimulated, IRF1-regulated cytokine release on tumor immunity and tumor growth. As Smac mimetics are currently evaluated in early clinical trials,^2^ a better understanding of Smac mimetic-initiated effects on tumor cells and their microenvironment has important implications for successful translation of this approach into clinical application.

## Materials and Methods

### Cell culture and chemicals

MDA-MB-231 breast carcinoma and SK-N-AS neuroblastoma cells were grown in DMEM medium (Invitrogen, Karlsruhe, Germany), T24 bladder carcinoma cells in McCoy's medium (Invitrogen) and Kym1 rhabdomyosarcoma cells in RPMI medium (Invitrogen) supplemented with 1% penicillin/streptomycin, 1% sodium pyruvate (both from Invitrogen) and 10% fetal calf serum (Invitrogen). The bivalent Smac mimetic BV6 that antagonizes XIAP, cIAP1 and cIAP2^6^ was kindly provided by Genentech Inc. (South San Francisco, CA, USA) and Enbrel by Pfizer (Berlin, Germany). Recombinant human TNF*α* was purchased from Biochrom (Berlin, Germany). All chemicals were obtained from Sigma (Deisenhofen, Germany) unless indicated otherwise.

### Transduction and siRNA transfection

Overexpression of the dominant-negative I*κ*B*α*-SR was performed by retroviral transduction as described previously.^43^ For transient knockdown by siRNA, cells were transfected with 5 nM Silencer Select siRNA (Invitrogen) control siRNA (no. 4390844) or targeting siRNAs (s7501, s7502 for IRF1; s14265, 14266 for TNFR1) using Lipofectamine RNAi Max (Invitrogen) and OptiMEM (Life Technologies, Carlsbad, CA, USA).

### Determination of apoptosis and cell viability

Apoptosis was determined by flow cytometric analysis of DNA fragmentation of PI-stained nuclei using FACSCanto II (BD Biosciences, Heidelberg, Germany) as described previously.^44^ The percentage of specific apoptosis was calculated as follows: % specific apoptosis=% induced apoptosis−% basal apoptosis. Cell viability was assessed by 3-(4,5-dimethylthiazol-2-yl)-2,5-diphenyltetrazolium bromide (MTT) assay according to the manufacturer's instructions (Roche Diagnostics, Mannheim, Germany).

### Western blotting and nuclear extraction

Western blot analysis was performed as described previously^45^ using the following antibodies: anti-*β*-actin (Sigma), anti-*α*-tubulin (Calbiochem, Darmstadt, Germany), anti-IRF1 and anti-TNFR1 (Santa Cruz Biotechnology, Santa Cruz, CA, USA) and anti-lamin A/C (Novacastra, Berlin, Germany). Donkey anti-mouse IgG or donkey anti-rabbit IgG labeled with IRDye infrared dyes were used for fluorescence detection at 700 nm 800 nm (LI-COR Biotechnology, Bad Homburg, Germany). IRF1 was immunodetected by enhanced chemoluminescence (Amersham Biosciences, Freiburg, Germany) using an anti-mouse IgG-HRP as the secondary antibody. Cytosolic and nuclear extracts were prepared as described previously.^46^ Briefly, for isolation of nuclear proteins, pelleted nuclei were resuspended in RIPA buffer, sonicated and nuclear supernatants were obtained by centrifugation.

### Quantitative RT-PCR

Total RNA was extracted using peqGOLD Total RNA kit from Peqlab Biotechnologie GmbH (Erlangen, Germany) according to the manufacturer's instructions. cDNA synthesis and quantification of gene expression were performed as described previously.^26^ Primers are listed in Supplementary Table 1. At least two independent experiments were performed for each gene. TNF*α* mRNA levels were assessed by TaqMan Gene Expression Assay (Life Technologies; Hs01113624_g1) according to the manufacturer's protocol.

### Determination of cytokine production

TNF*α* protein levels in cell culture supernatants were measured using TNF*α* human ultrasensitive ELISA Kit from Life Technologies according to the manufacturer's instructions. GM-CSF, IL-6, IL-8 and MCP-1 protein levels in cell supernatants were quantified using Cytometric Bead Array (CBA) Flex Sets, an LSRFortessa flow cytometer and FCAP software (all from BD Biosciences) following the manufacturer's instructions. Cells were seeded in 6-well plates and treated with BV6 in 1.25 ml medium for 15 h (TNF*α* ELISA) or 1.25 ml medium supplemented 20 *μ*M zVAD.fmk (Z-Val-Ala-Asp (OMe)-fluoromethyl ketone) for 15 h (CBA). Cell culture supernatant was centrifuged at 4 °C for 20 min, snap-frozen in liquid nitrogen and stored at −80 °C or directly subjected to cytokine measurement.

### NF-*κ*B reporter assay

NF-*κ*B transcriptional activity was determined using the NF-*κ*B Reporter System pTRH1-NF-*κ*B-EGFP from System Biosciences (Mountain View, CA, USA; no. TR503PA-1) according to the manufacturer's instructions. Briefly, NF-*κ*B transcriptional activity was determined by flow cytometric analysis of median FITC intensity of the living cell population as described previously.^26^

### Migration assay

MDA-MB-231 cells were seeded in 24-well plates, treated with 50 nM BV6 for 3 h before the medium was replaced by drug-free and reduced serum (0.1%) medium supplemented with 20 *μ*M zVAD.fmk and incubated overnight. Thereafter, 10^6^ freshly isolated PBMCs from healthy donors were applied in transwell inserts and allowed to migrate towards MDA-MB-231 cells in the lower compartment. The number of migrated monocytes was determined after 3 h using an LSRFortessa flow cytometer. Monocytes were identified as CD45+, CD3−, CD19−, CD14+ cells, and Flow-Count fluorospheres (Beckman Coulter, Krefeld, Germany) were used as an internal counting standard in each tube. The FlowJo 7.6.5 software (Treestar, Ashland, OR, USA) was used for data analysis.

### Statistical analysis

Statistical significance was assessed by two-sided Student's *t*-test using Microsoft Excel (Microsoft Deutschland GmbH, Unterschleißheim, Germany).

## Abbreviations

CBA: cytometric bead assay
CHX: cycloheximide
cIAP1: cellular inhibitor of apoptosis
GM-CSF: granulocyte–macrophage colony-stimulating factor
IAP: inhibitor of apoptosis
IFN: interferon
IL-8: interleukin-8
IRF: interferon regulatory factor
I*κ*B*α*-SR: I*κ*B*α* superrepressor
LPS: lipopolysaccharide
MCP-1: monocyte chemoattractant protein-1
MTT: 3-(4,5-dimethylthiazol-2-yl)-2,5-diphenyltetrazolium bromide
NF-*κ*B: nuclear factor-*κ*B
NK: natural killer
PI: propidium iodide
RIP1: receptor-interacting protein 1
qRT-PCR: quantitative reverse transcriptase PCR
Smac: second mitochondria-derived activator of caspase
TNF: tumor necrosis factor
TNFR1: tumor necrosis factor receptor 1
TRAF: TNF receptor-associated factor
XIAP: X-linked inhibitor of apoptosis protein
zVAD-FMK: Z-Val-Ala-Asp (OMe)-fluoromethyl ketone

## References

[bib1] 1Hanahan D, Weinberg RA. Hallmarks of cancer: the next generation. Cell 2011; 144: 646–674. 21376230 10.1016/j.cell.2011.02.013

[bib2] 2Fulda S, Vucic D. Targeting IAP proteins for therapeutic intervention in cancer. Nat Rev Drug Discov 2012; 11: 109–124. 22293567 10.1038/nrd3627

[bib3] 3Bertrand MJ, Milutinovic S, Dickson KM, Ho WC, Boudreault A, Durkin J et al. cIAP1 and cIAP2 facilitate cancer cell survival by functioning as E3 ligases that promote RIP1 ubiquitination. Mol Cell 2008; 30: 689–700. 18570872 10.1016/j.molcel.2008.05.014

[bib4] 4Hayden MS, Ghosh S. Shared principles in NF-kappaB signaling. Cell 2008; 132: 344–362. 18267068 10.1016/j.cell.2008.01.020

[bib5] 5Zarnegar BJ, Wang Y, Mahoney DJ, Dempsey PW, Cheung HH, He J et al. Noncanonical NF-kappaB activation requires coordinated assembly of a regulatory complex of the adaptors cIAP1, cIAP2, TRAF2 and TRAF3 and the kinase NIK. Nat Immunol 2008; 9: 1371–1378. 18997794 10.1038/ni.1676 PMC2676931

[bib6] 6Varfolomeev E, Blankenship JW, Wayson SM, Fedorova AV, Kayagaki N, Garg P et al. IAP antagonists induce autoubiquitination of c-IAPs, NF-kappaB activation, and TNFalpha-dependent apoptosis. Cell 2007; 131: 669–681. 18022362 10.1016/j.cell.2007.10.030

[bib7] 7Vince JE, Wong WW, Khan N, Feltham R, Chau D, Ahmed AU et al. IAP antagonists target cIAP1 to induce TNFalpha-dependent apoptosis. Cell 2007; 131: 682–693. 18022363 10.1016/j.cell.2007.10.037

[bib8] 8Savitsky D, Tamura T, Yanai H, Taniguchi T. Regulation of immunity and oncogenesis by the IRF transcription factor family. Cancer Immunol Immunother 2010; 59: 489–510. 20049431 10.1007/s00262-009-0804-6 PMC11030943

[bib9] 9Miyamoto M, Fujita T, Kimura Y, Maruyama M, Harada H, Sudo Y et al. Regulated expression of a gene encoding a nuclear factor, IRF-1, that specifically binds to IFN-beta gene regulatory elements. Cell 1988; 54: 903–913. 3409321 10.1016/s0092-8674(88)91307-4

[bib10] 10Nakagawa K, Yokosawa H. Degradation of transcription factor IRF-1 by the ubiquitin-proteasome pathway. The C-terminal region governs the protein stability. Eur J Biochem/FEBS 2000; 267: 1680–1686. 10.1046/j.1432-1327.2000.01163.x 10712599

[bib11] 11Moschonas A, Kouraki M, Knox PG, Thymiakou E, Kardassis D, Eliopoulos AG. CD40 induces antigen transporter and immunoproteasome gene expression in carcinomas via the coordinated action of NF-kappaB and of NF-kappaB-mediated *de novo* synthesis of IRF-1. Mol Cell Biol 2008; 28: 6208–6222. 18694960 10.1128/MCB.00611-08 PMC2577429

[bib12] 12Andersen P, Pedersen MW, Woetmann A, Villingshoj M, Stockhausen MT, Odum N et al. EGFR induces expression of IRF-1 via STAT1 and STAT3 activation leading to growth arrest of human cancer cells. Int J Cancer 2008; 122: 342–349. 17918184 10.1002/ijc.23109

[bib13] 13Lehtonen A, Matikainen S, Julkunen I. Interferons up-regulate STAT1, STAT2, and IRF family transcription factor gene expression in human peripheral blood mononuclear cells and macrophages. J Immunol 1997; 159: 794–803. 9218597

[bib14] 14Taniguchi T, Ogasawara K, Takaoka A, Tanaka N. IRF family of transcription factors as regulators of host defense. Annu Rev Immunol 2001; 19: 623–655. 11244049 10.1146/annurev.immunol.19.1.623

[bib15] 15Pamment J, Ramsay E, Kelleher M, Dornan D, Ball KL. Regulation of the IRF-1 tumour modifier during the response to genotoxic stress involves an ATM-dependent signalling pathway. Oncogene 2002; 21: 7776–7785. 12420214 10.1038/sj.onc.1205981

[bib16] 16Bouker KB, Skaar TC, Riggins RB, Harburger DS, Fernandez DR, Zwart A et al. Interferon regulatory factor-1 (IRF-1) exhibits tumor suppressor activities in breast cancer associated with caspase activation and induction of apoptosis. Carcinogenesis 2005; 26: 1527–1535. 15878912 10.1093/carcin/bgi113

[bib17] 17Clarke N, Jimenez-Lara AM, Voltz E, Gronemeyer H. Tumor suppressor IRF-1 mediates retinoid and interferon anticancer signaling to death ligand TRAIL. EMBO J 2004; 23: 3051–3060. 15241475 10.1038/sj.emboj.7600302 PMC514919

[bib18] 18Gao J, Senthil M, Ren B, Yan J, Xing Q, Yu J et al. IRF-1 transcriptionally upregulates PUMA, which mediates the mitochondrial apoptotic pathway in IRF-1-induced apoptosis in cancer cells. Cell Death Differ 2010; 17: 699–709. 19851330 10.1038/cdd.2009.156 PMC2838929

[bib19] 19Lee JH, Chun T, Park SY, Rho SB. Interferon regulatory factor-1 (IRF-1) regulates VEGF-induced angiogenesis in HUVECs. Biochim Biophys Acta 2008; 1783: 1654–1662. 18472010 10.1016/j.bbamcr.2008.04.006

[bib20] 20Kollet JI, Petro TM. IRF-1 and NF-kappaB p50/cRel bind to distinct regions of the proximal murine IL-12 p35 promoter during costimulation with IFN-gamma and LPS. Mol Immunol 2006; 43: 623–633. 15871905 10.1016/j.molimm.2005.04.004

[bib21] 21Sgarbanti M, Remoli AL, Marsili G, Ridolfi B, Borsetti A, Perrotti E et al. IRF-1 is required for full NF-kappaB transcriptional activity at the human immunodeficiency virus type 1 long terminal repeat enhancer. J Virol 2008; 82: 3632–3641. 18216101 10.1128/JVI.00599-07 PMC2268499

[bib22] 22Wagner L, Marschall V, Karl S, Cristofanon S, Zobel K, Deshayes K et al. Smac mimetic sensitizes glioblastoma cells to Temozolomide-induced apoptosis in a RIP1- and NF-kappaB-dependent manner. Oncogene 2013; 32: 988–997. 22469979 10.1038/onc.2012.108

[bib23] 23Stadel D, Cristofanon S, Abhari BA, Deshayes K, Zobel K, Vucic D et al. Requirement of nuclear factor kappaB for Smac mimetic-mediated sensitization of pancreatic carcinoma cells for gemcitabine-induced apoptosis. Neoplasia 2011; 13: 1162–1170. 22241962 10.1593/neo.11460 PMC3257191

[bib24] 24Berger R, Jennewein C, Marschall V, Karl S, Cristofanon S, Wagner L et al. NF-{kappa}B is required for smac mimetic-mediated sensitization of glioblastoma cells for {gamma}-irradiation-induced apoptosis. Mol Cancer Ther 2011; 10: 1867–1875. 21859841 10.1158/1535-7163.MCT-11-0218

[bib25] 25Kim PK, Armstrong M, Liu Y, Yan P, Bucher B, Zuckerbraun BS et al. IRF-1 expression induces apoptosis and inhibits tumor growth in mouse mammary cancer cells *in vitro* and *in vivo*. Oncogene 2004; 23: 1125–1135. 14762441 10.1038/sj.onc.1207023

[bib26] 26Eckhardt I, Roesler S, Fulda S. Identification of DR5 as a critical, NF-kappaB-regulated mediator of Smac-induced apoptosis. Cell Death Dis 2013; 4: e936. 24287697 10.1038/cddis.2013.457 PMC3847333

[bib27] 27Tchoghandjian A, Jennewein C, Eckhardt I, Rajalingam K, Fulda S. Identification of non-canonical NF-κB signaling as a critical mediator of Smac mimetic-stimulated migration and invasion of glioblastoma cells. Cell Death Dis 2013; 4: e564. 23538445 10.1038/cddis.2013.70 PMC3615728

[bib28] 28Tchoghandjian A, Jennewein C, Eckhardt I, Momma S, Figarella-Branger D, Fulda S. Smac mimetic promotes glioblastoma cancer stem-like cell differentiation by activating NF-κB. Cell Death Differ 2014; 21: 735–747. 24488095 10.1038/cdd.2013.200 PMC3978303

[bib29] 29Krausgruber T, Saliba D, Ryzhakov G, Lanfrancotti A, Blazek K, Udalova IA. IRF5 is required for late-phase TNF secretion by human dendritic cells. Blood 2010; 115: 4421–4430. 20237317 10.1182/blood-2010-01-263020

[bib30] 30Tamura G, Ogasawara S, Nishizuka S, Sakata K, Maesawa C, Suzuki Y et al. Two distinct regions of deletion on the long arm of chromosome 5 in differentiated adenocarcinomas of the stomach. Cancer Res 1996; 56: 612–615. 8564980

[bib31] 31Boultwood J, Fidler C, Lewis S, MacCarthy A, Sheridan H, Kelly S et al. Allelic loss of IRF1 in myelodysplasia and acute myeloid leukemia: retention of IRF1 on the 5q− chromosome in some patients with the 5q− syndrome. Blood 1993; 82: 2611–2616. 8219215

[bib32] 32Cavalli LR, Riggins RB, Wang A, Clarke R, Haddad BR. Frequent loss of heterozygosity at the interferon regulatory factor-1 gene locus in breast cancer. Breast Cancer Res Treat 2010; 121: 227–231. 19697121 10.1007/s10549-009-0509-8 PMC2941871

[bib33] 33Ogasawara S, Tamura G, Maesawa C, Suzuki Y, Ishida K, Satoh N et al. Common deleted region on the long arm of chromosome 5 in esophageal carcinoma. Gastroenterology 1996; 110: 52–57. 8536888 10.1053/gast.1996.v110.pm8536888

[bib34] 34Vila-del Sol V, Punzon C, Fresno M. IFN-gamma-induced TNF-alpha expression is regulated by interferon regulatory factors 1 and 8 in mouse macrophages. J Immunol 2008; 181: 4461–4470. 18802049 10.4049/jimmunol.181.7.4461

[bib35] 35Zhao XJ, Dong Q, Bindas J, Piganelli JD, Magill A, Reiser J et al. TRIF and IRF-3 binding to the TNF promoter results in macrophage TNF dysregulation and steatosis induced by chronic ethanol. J Immunol 2008; 181: 3049–3056. 18713975 10.4049/jimmunol.181.5.3049 PMC3690475

[bib36] 36Kearney CJ, Sheridan C, Cullen SP, Tynan GA, Logue SE, Afonina IS et al. Inhibitor of apoptosis proteins (IAPs) and their antagonists regulate spontaneous and tumor necrosis factor (TNF)-induced proinflammatory cytokine and chemokine production. J Biol Chem 2013; 288: 4878–4890. 23275336 10.1074/jbc.M112.422410 PMC3576092

[bib37] 37Ksienzyk A, Neumann B, Nandakumar R, Finsterbusch K, Grashoff M, Zawatzky R et al. IRF-1 expression is essential for natural killer cells to suppress metastasis. Cancer Res 2011; 71: 6410–6418. 21900395 10.1158/0008-5472.CAN-11-1565

[bib38] 38Emeagi PU, Van Lint S, Goyvaerts C, Maenhout S, Cauwels A, McNeish IA et al. Proinflammatory characteristics of SMAC/DIABLO-induced cell death in antitumor therapy. Cancer Res 2012; 72: 1342–1352. 22379024 10.1158/0008-5472.CAN-11-2400

[bib39] 39Hu Y, Park-Min KH, Yarilina A, Ivashkiv LB. Regulation of STAT pathways and IRF1 during human dendritic cell maturation by TNF-alpha and PGE2. J Leukoc Biol 2008; 84: 1353–1360. 18678606 10.1189/jlb.0107040 PMC2567899

[bib40] 40Landre V, Pion E, Narayan V, Xirodimas DP, Ball KL. DNA-binding regulates site-specific ubiquitination of IRF-1. Biochem J 2013; 449: 707–717. 23134341 10.1042/BJ20121076

[bib41] 41Shen Y, Xia M, Zhang J, Xu L, Yang J, Chen A et al. IRF-1 and p65 mediate upregulation of constitutive HLA-A antigen expression by hepatocellular carcinoma cells. Mol Immunol 2009; 46: 2045–2053. 19428110 10.1016/j.molimm.2009.03.001 PMC3426235

[bib42] 42Mantovani A, Allavena P, Sica A, Balkwill F. Cancer-related inflammation. Nature 2008; 454: 436–444. 18650914 10.1038/nature07205

[bib43] 43Karl S, Pritschow Y, Volcic M, Hacker S, Baumann B, Wiesmuller L et al. Identification of a novel pro-apopotic function of NF-kappaB in the DNA damage response. J Cell Mol Med 2009; 13: 4239–4256. 19725919 10.1111/j.1582-4934.2009.00888.x PMC4496130

[bib44] 44Fulda S, Friesen C, Los M, Scaffidi C, Mier W, Benedict M et al. Betulinic acid triggers CD95 (APO-1/Fas)- and p53-independent apoptosis via activation of caspases in neuroectodermal tumors. Cancer Res 1997; 57: 4956–4964. 9354463

[bib45] 45Vogler M, Durr K, Jovanovic M, Debatin KM, Fulda S. Regulation of TRAIL-induced apoptosis by XIAP in pancreatic carcinoma cells. Oncogene 2007; 26: 248–257. 16832350 10.1038/sj.onc.1209776

[bib46] 46Kasperczyk H, La Ferla-Brühl K, Westhoff MA, Behrend L, Zwacka RM, Debatin KM et al. Betulinic acid as new activator of NF-kappaB: molecular mechanisms and implications for cancer therapy. Oncogene 2005; 24: 6945–6956. 16007147 10.1038/sj.onc.1208842

