# Supplementary material for: Identification of IRF1 as critical dual regulator of Smac mimetic-induced apoptosis and inflammatory cytokine response
Source: Cell Death Dis. 2014 Dec 11;5(12):e1562–. doi: 10.1038/cddis.2014.498 (PMC4454156; doi:10.1038/cddis.2014.498)
Supplement: Supplementary Information [file cddis2014498x1.pdf]

a

sample	mean IRF1 signal intensity
control	481.25
control + BV6	1053.50
IκBα-SR	282.95
IκBα-SR + BV6	313.68

b

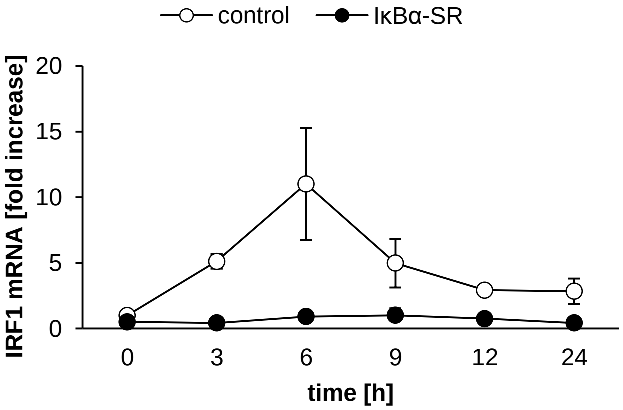

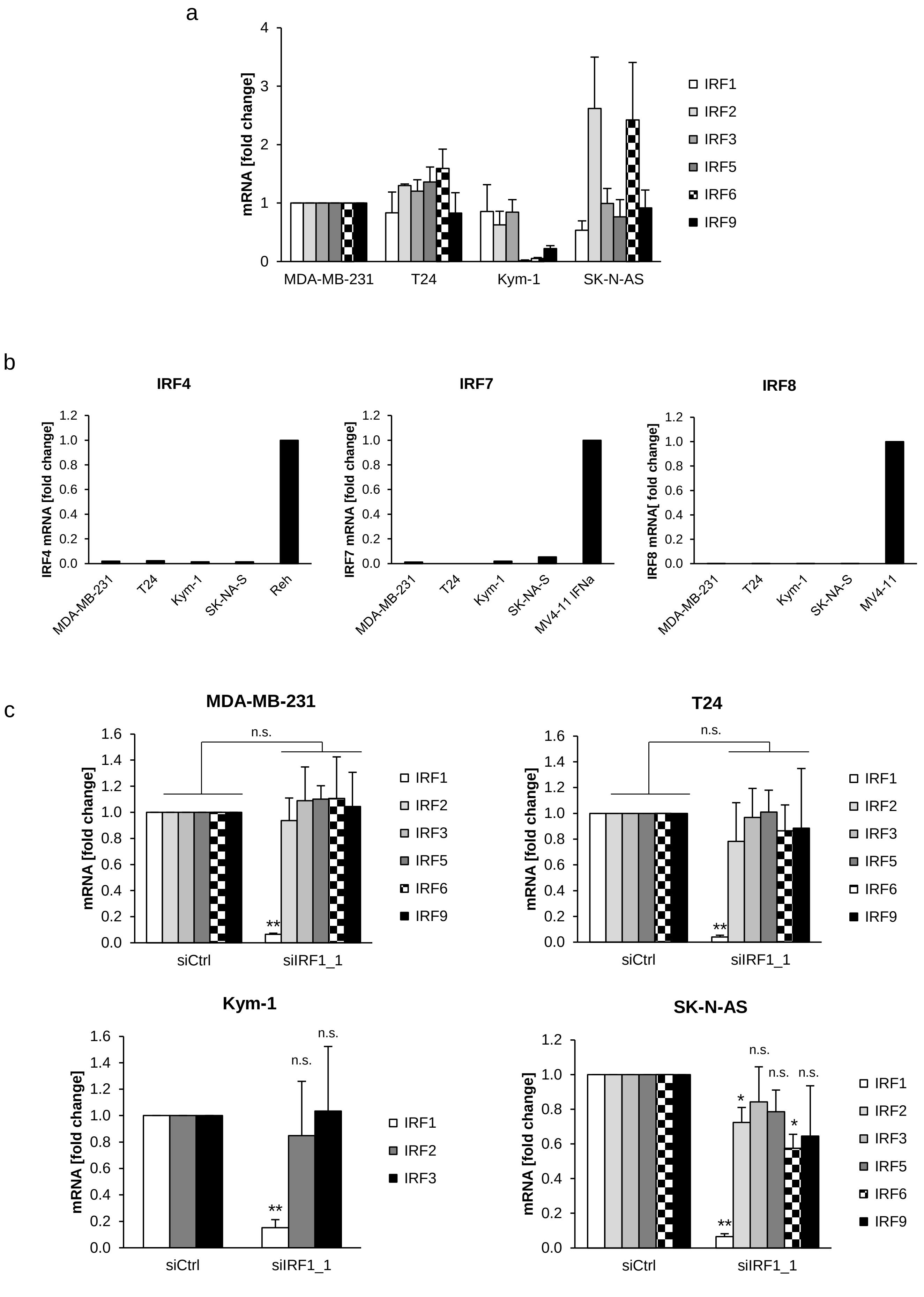
a
mRNA [fold change]
IRF1
IRF2
IRF3
IRF5
IRF6
IRF9
MDA-MB-231
T24
Kym-1
SK-N-AS
b
IRF4
IRF7
IRF8
IRF4 mRNA [fold change]
IRF7 mRNA [fold change]
IRF8 mRNA[fold change]
MDA-MB-231
T24
Kym-1
SK-NA-S
Reh
MV4-11 IFNa
MV4-11
c
MDA-MB-231
T24
Kym-1
SK-N-AS
n.s.
*
**
siCtrl
siIRF1_1

MDA-MB-231

a

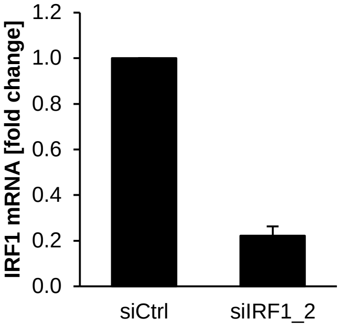

b

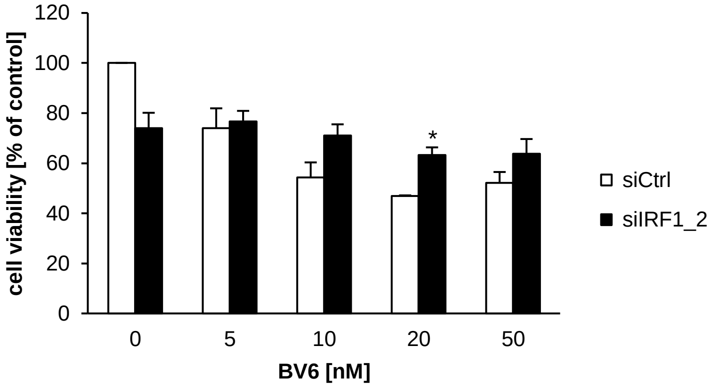

c

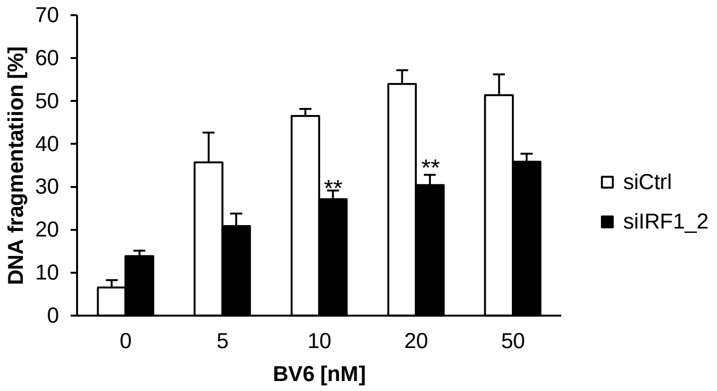

Suppl. Figure 4

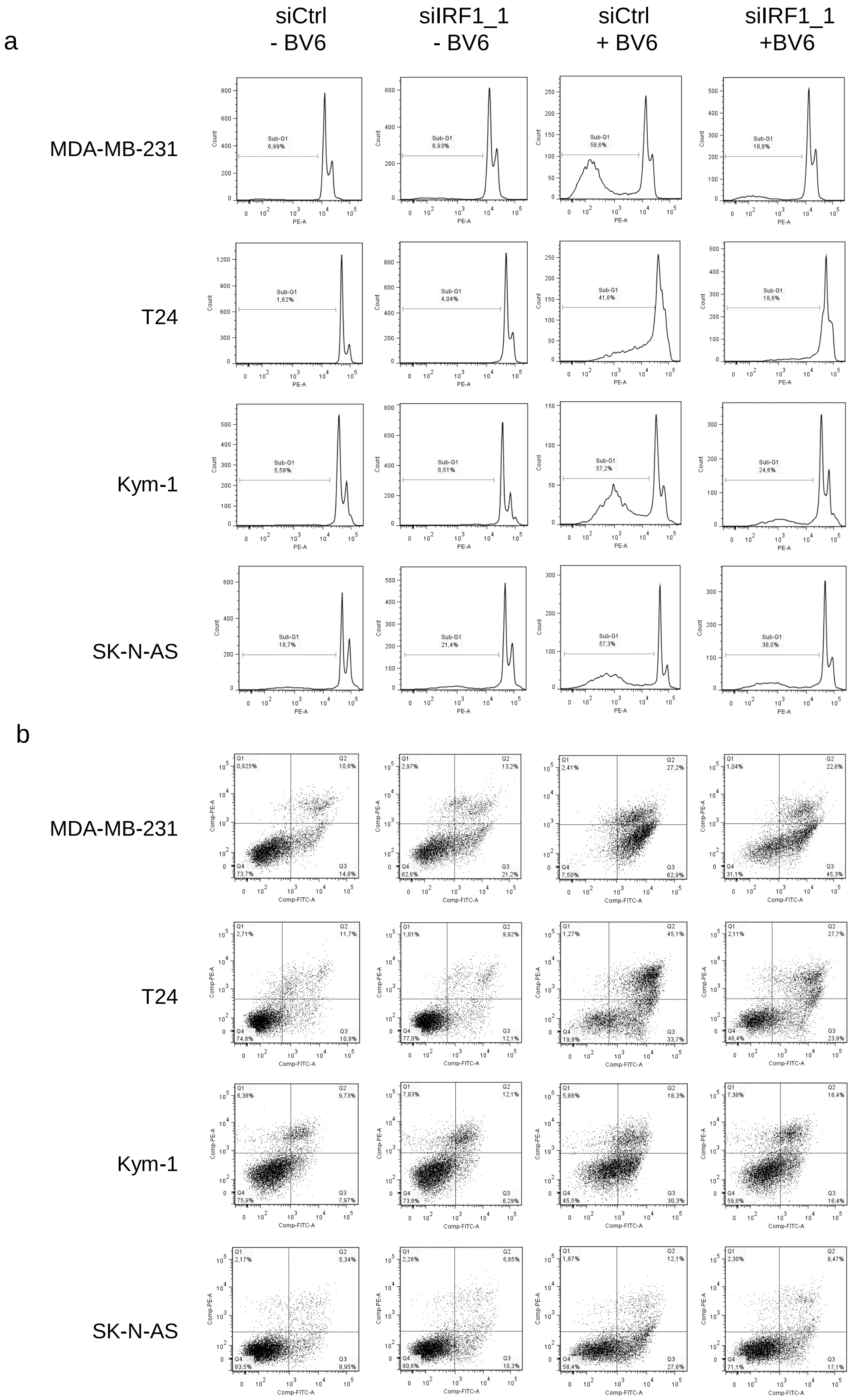

Suppl. Figure 5

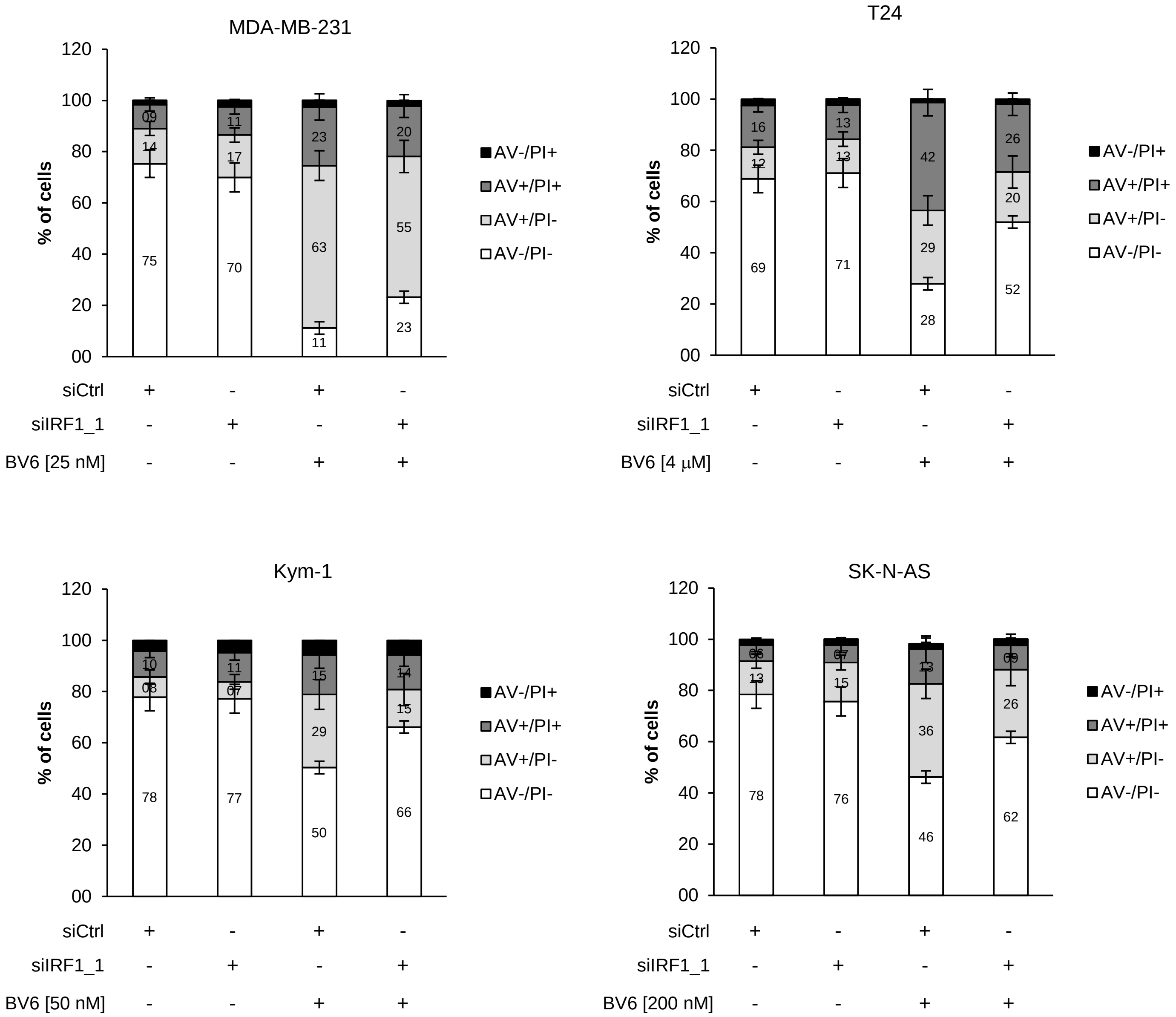

a

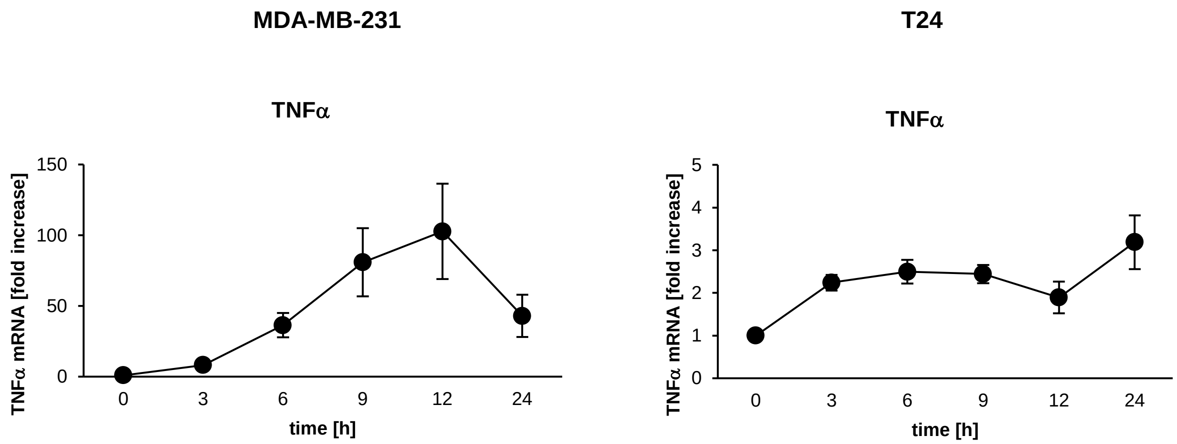
MDA-MB-231
T24
TNFα
TNFα
TNFα mRNA [fold increase]
150
100
50
0
0
3
6
9
12
24
time [h]
TNFα mRNA [fold increase]
5
4
3
2
1
0
0
3
6
9
12
24
time [h]

b

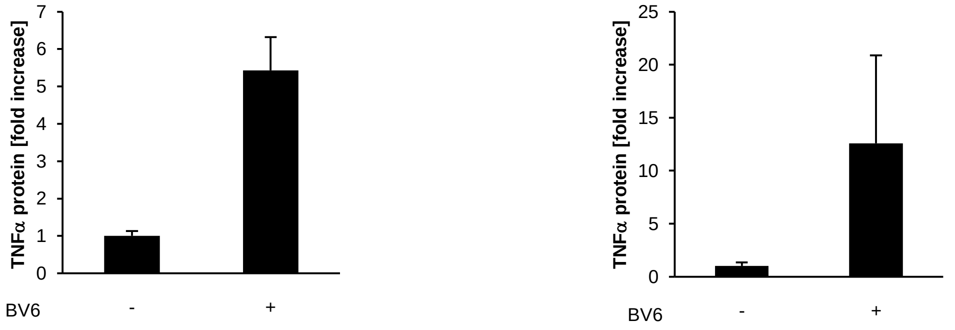
TNFα protein [fold increase]
7
6
5
4
3
2
1
0
BV6
-
+
TNFα protein [fold increase]
25
20
15
10
5
0
BV6
-
+

Suppl. Figure 7

T24

a

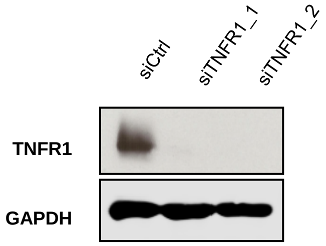

b

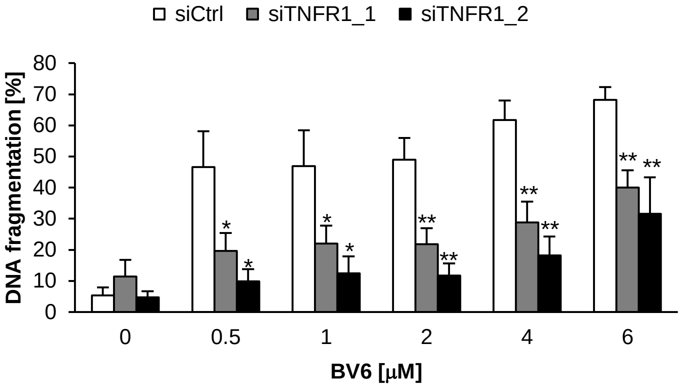

Suppl. Figure 8

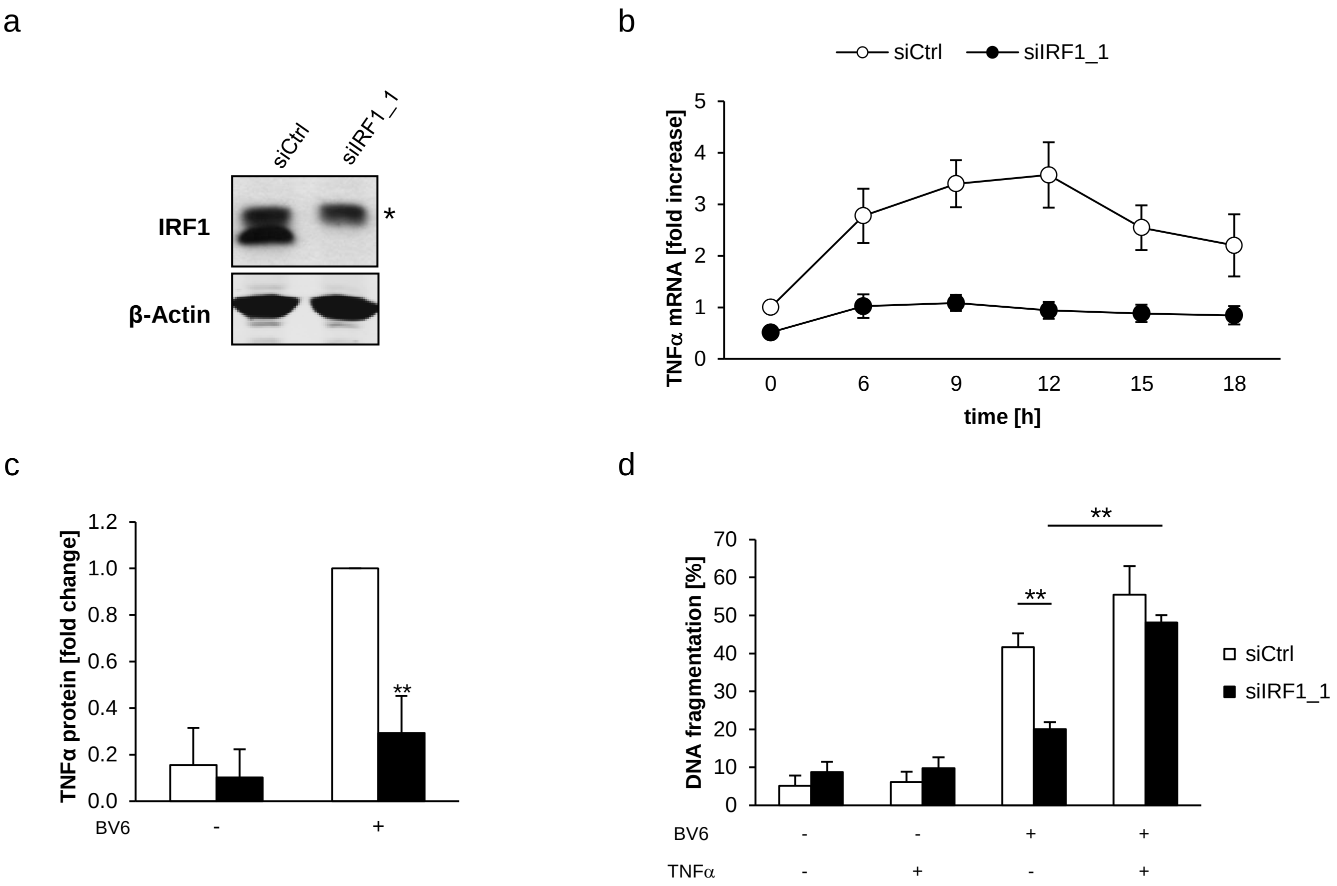

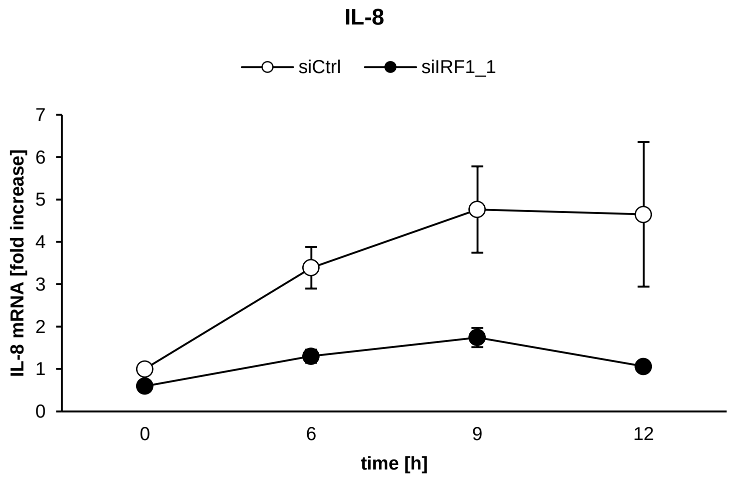
IL-8
siCtrl
siIRF1_1
IL-8 mRNA [fold increase]
0
1
2
3
4
5
6
7
0
6
9
12
time [h]

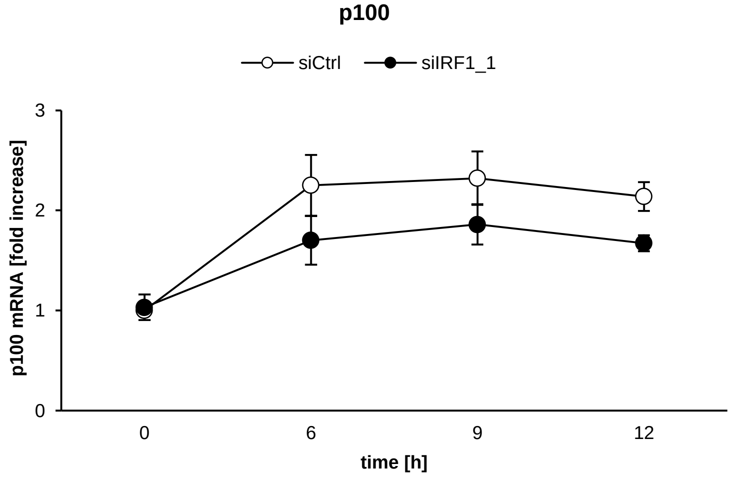
p100
siCtrl
siIRF1_1
p100 mRNA [fold increase]
0
1
2
3
0
6
9
12
time [h]

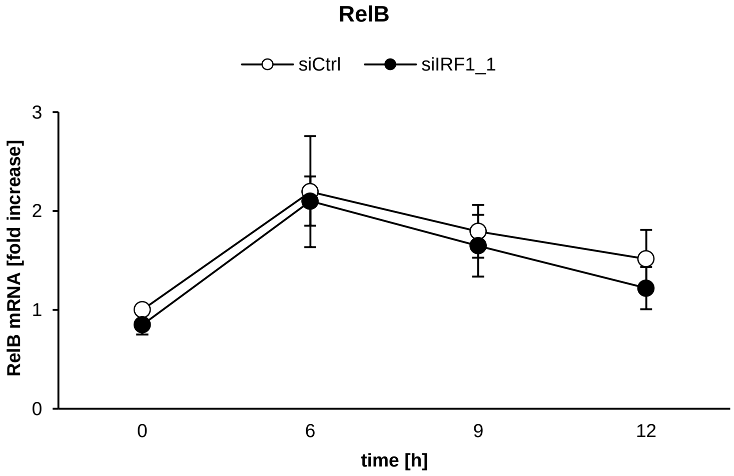
RelB
siCtrl
siIRF1_1
RelB mRNA [fold increase]
0
1
2
3
0
6
9
12
time [h]

a

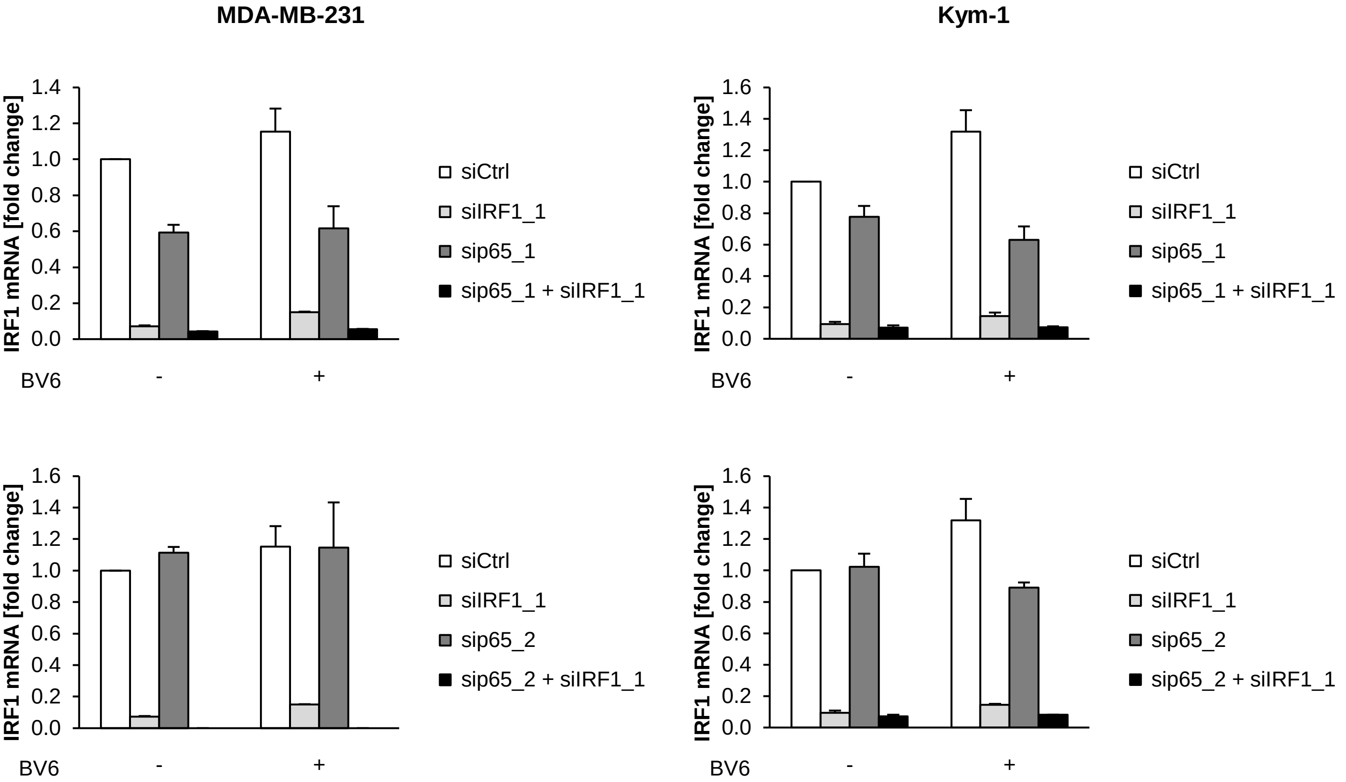
MDA-MB-231
Kym-1
IRF1 mRNA [fold change]
BV6
-
+
siCtrl
siIRF1_1
sip65_1
sip65_1 + siIRF1_1
sip65_2
sip65_2 + siIRF1_1

b

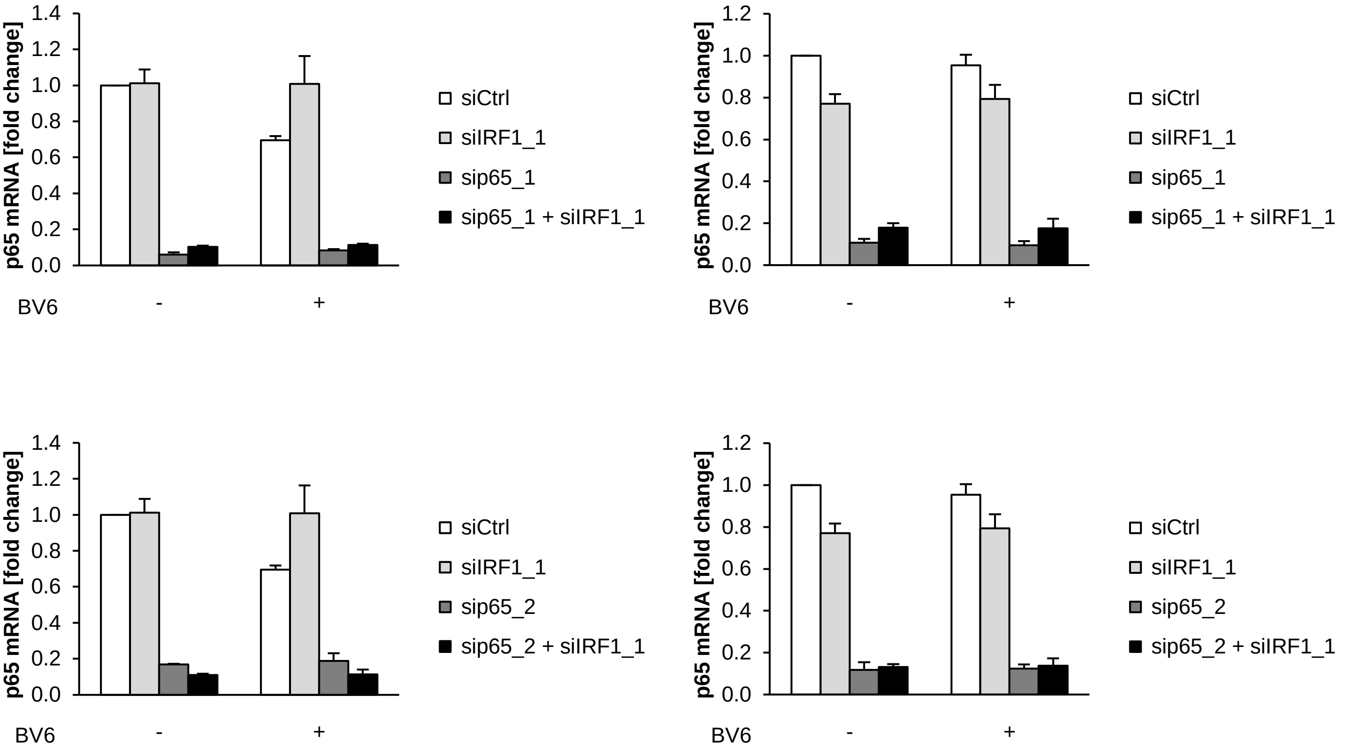
p65 mRNA [fold change]
BV6
-
+
siCtrl
siIRF1_1
sip65_1
sip65_1 + siIRF1_1
sip65_2
sip65_2 + siIRF1_1

a

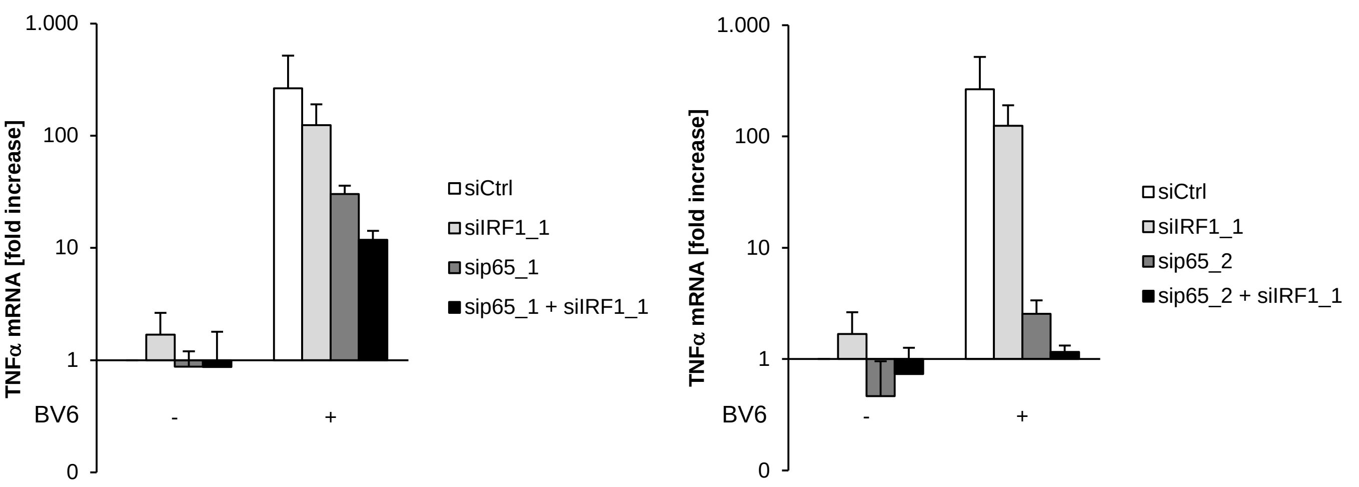

TNFα mRNA [fold increase]
1.000
100
10
1
0
BV6
-
+
siCtrl
siIRF1_1
sip65_1
sip65_1 + siIRF1_1
sip65_2
sip65_2 + siIRF1_1

b

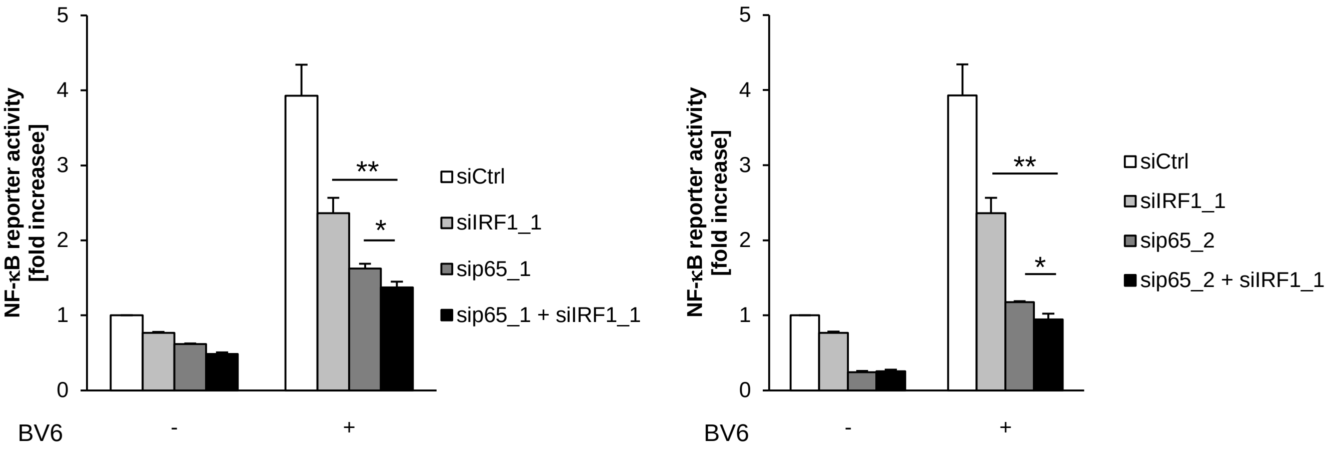

NF-κB reporter activity [fold increasee]
NF-κB reporter activity [fold increase]
5
4
3
2
1
0
BV6
-
+
**
*
siCtrl
siIRF1_1
sip65_1
sip65_1 + siIRF1_1
sip65_2
sip65_2 + siIRF1_1

a

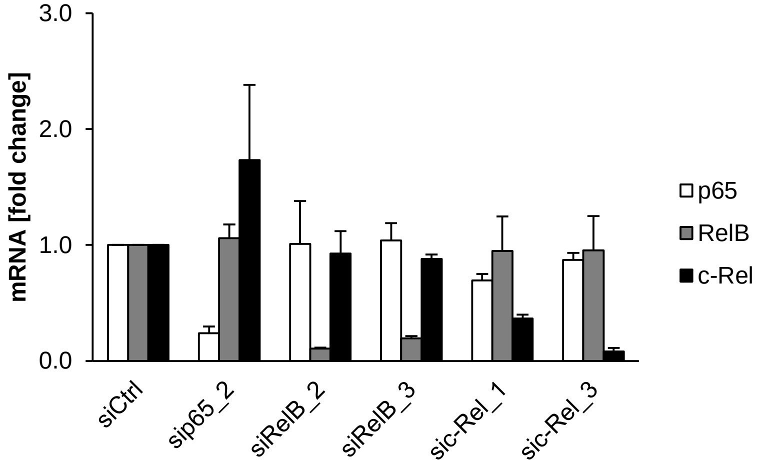
3.0
2.0
1.0
0.0
mRNA [fold change]
p65
RelB
c-Rel
siCtrl
sip65_2
siRelB_2
siRelB_3
sic-Rel_1
sic-Rel_3

b

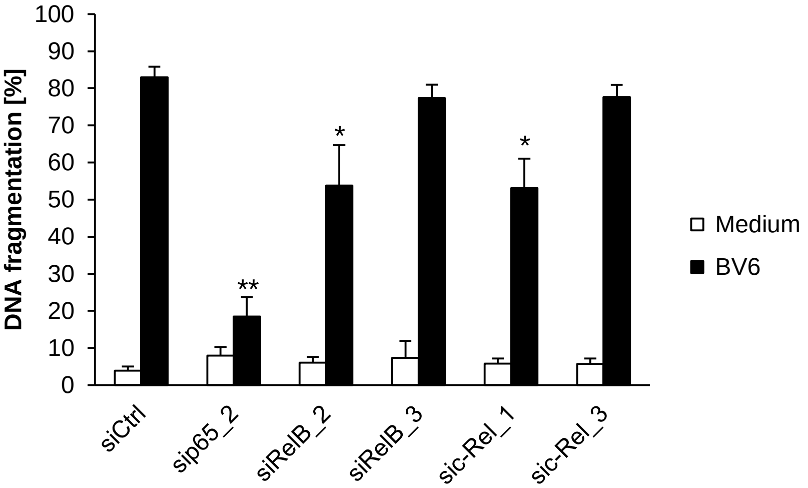
100
90
80
70
60
50
40
30
20
10
0
DNA fragmentation [%]
**
*
*
Medium
BV6
siCtrl
sip65_2
siRelB_2
siRelB_3
sic-Rel_1
sic-Rel_3

Suppl. Figure 13

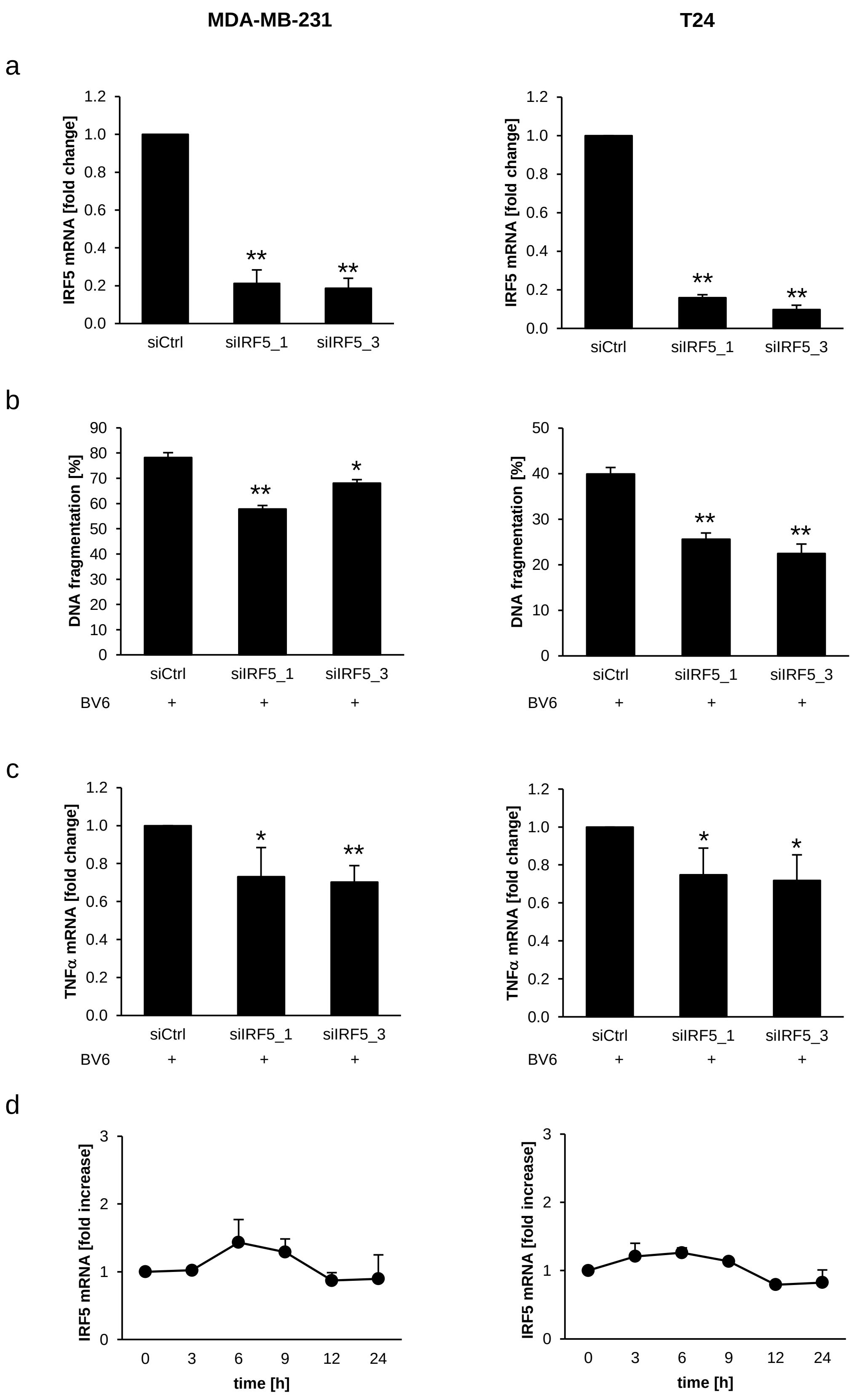

Suppl. Figure 14

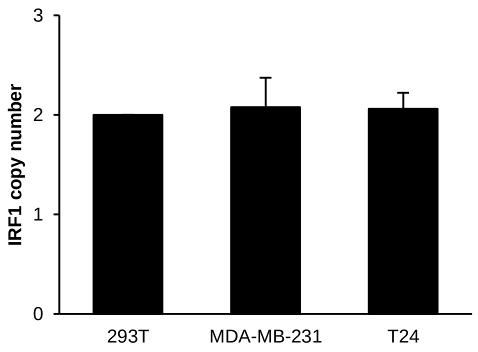

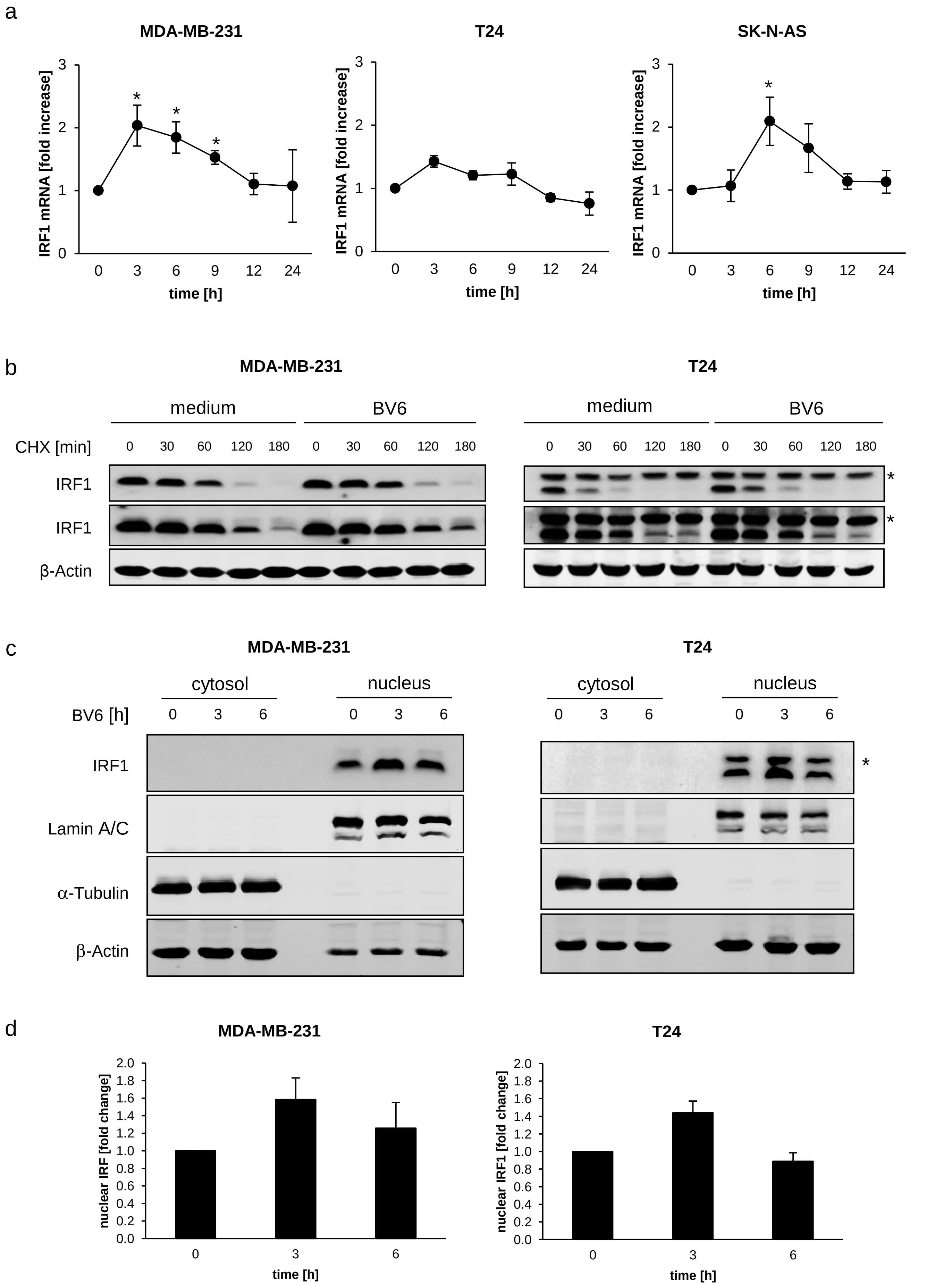
a
MDA-MB-231
T24
SK-N-AS
IRF1 mRNA [fold increase]
time [h]
0
3
6
9
12
24
b
MDA-MB-231
T24
medium
BV6
CHX [min]
0
30
60
120
180
IRF1
β-Actin
c
cytosol
nucleus
BV6 [h]
IRF1
Lamin A/C
α-Tubulin
β-Actin
d
nuclear IRF [fold change]
nuclear IRF1 [fold change]
time [h]

Supplemental Tab. 1 Primer Sequences

Target	Forward primer	Reverse primer
**28s rRNA**	ttgaaaatccgggggagag	acattgttccaacatgccag
**IRF1**	acagcaccagtgatctgtacaac	ttcccttcctcatcctcatct
**IRF2**	tgaagtggatagtacggtgaaca	cggattggtgacaatctcttg
**IRF3**	aaggaaggaggcgtgtttg	ttccttccgtgaaggtaatca
**IRF4**	gccaagattccaggtgactc	ctggctagcagaggttctacg
**IRF5**	tctacgaggtctgctccaatg	catcctctgcagctcttcct
**IRF6**	ggcatagccctcaacaagaa	caccccttcctggtacttcc
**IRF7**	aagggcttcccctgactg	tctactgcccacccgtaca
**IRF8**	gaggtggtccaggtcttcg	cggccctggctgttatag
**IRF9**	agcctggacagcaactcag	gaaactgcccactctccact
**p100**	cacatgggtggaggctct	actggtaggggctgtaggc
**RelA**	tcatgaagaagagtcctttcagc	ggatgacgtaaagggataggg
**RelB**	gctctacttgctctgcagaca	ggcctgggagaagtcagc
**cRel**	tgaacatggtaatttgacgactg	acacgacaaatccttaattctgc
**Interleukin-8**	ctcttggcagccttcctgatt	tatgcactgacatctaagttctttagca

Supplemental Tab. 2 Microarray results

Gene Symbol	Ratio CV BV6/CV DMSO	p value	Ratio SR BV6/SR DMSO	p value
CCL2	9.17	1.75E-48	0.95	5.36E-01
BIRC3	5.30	2.76E-40	0.92	2.55E-01
VCAM1	4.68	3.34E-37	1.05	4.79E-01
IRAK2	3.63	3.84E-31	1.28	4.73E-04
TRAF1	2.63	7.45E-23	0.94	5.42E-01
NFKBIA	2.57	5.18E-34	0.90	4.29E-03
NFKB2	2.34	1.78E-43	1.01	8.97E-01
SOX11	2.28	9.30E-23	1.15	8.28E-05
NFKBIE	2.22	3.50E-49	0.95	5.91E-01
**IRF1**	**2.19**	**2.66E-21**	**1.11**	**8.76E-02**
BCL3	2.15	2.17E-47	0.90	3.07E-02
MTSS1	2.14	1.63E-26	1.08	3.25E-01
SSTR2	2.12	1.06E-18	1.03	8.08E-01
TNFAIP2	2.08	3.69E-23	1.02	8.17E-01
IL4I1	2.08	1.06E-15	1.03	7.90E-01
TNIP1	2.03	1.49E-32	1.00	9.87E-01
GOLSYN	0.50	3.33E-31	0.83	1.22E-04
TNFSF4	1.97	2.56E-20	0.96	4.81E-01
DIO2	0.51	1.10E-17	0.77	1.08E-05
BIRC2	1.89	1.71E-14	1.05	5.81E-01
NAV2	1.82	1.10E-23	1.07	2.47E-01
CD83	1.82	1.70E-28	1.06	4.30E-01
LRIG1	1.80	4.40E-22	1.09	1.47E-01
RELB	1.78	2.10E-25	0.99	9.31E-01
SLC25A22	1.74	6.50E-16	1.06	2.61E-01
TMEM51	1.72	1.16E-18	1.22	1.18E-05
BDKRB1	1.71	5.79E-26	0.98	8.70E-01
TNFAIP3	1.69	1.05E-18	1.13	2.23E-01
TMEM194A	1.68	2.81E-16	1.03	7.32E-01
ZNF697	1.68	6.63E-14	1.25	8.78E-06
LARP6	1.68	7.07E-16	1.33	1.45E-06
CD70	1.68	4.23E-15	0.96	5.47E-01
LOC728855	1.66	2.04E-19	1.09	2.42E-01
SLFN11	1.65	3.25E-18	1.15	2.51E-02
TM4SF1	1.63	9.16E-19	1.23	7.49E-05
TNFRSF10B	1.62	1.13E-12	1.14	7.46E-03
TMEM194A	1.62	1.02E-16	1.12	3.54E-02
NXPH2	0.62	1.84E-29	0.83	4.17E-06
ZBTB46	1.62	6.73E-31	1.13	3.36E-03
STAT5A	1.60	2.71E-11	1.12	1.90E-01
NFKB1	1.60	3.01E-20	1.03	6.01E-01
RNF19B	1.59	9.48E-17	1.15	1.21E-02
C1QTNF1	1.58	8.37E-15	0.94	3.97E-01
S1PR3	1.58	1.15E-15	1.06	5.18E-01
ZSWIM4	1.58	3.00E-22	1.08	1.26E-01
GYPC	1.57	7.41E-19	1.06	3.58E-01
FOXA2	1.56	1.42E-25	1.14	1.72E-03
TLN2	0.64	2.28E-24	0.93	2.37E-01
DUSP5	1.56	2.01E-13	1.20	6.66E-04
IL27RA	1.55	1.73E-21	1.07	2.31E-03

Top 50 regulated genes in Whole genome expression array

Genes were ranked according to their fold regulation in untreated vs treated control vector (CV) cells. Genes that were similarly regulated in IκBα-SR (SR) overexpressing cells were removed from the analysis. Regulation with a p-value of $<1.25^{-6}$ were considered as significant. IRF1 ranked number 10 in this analysis.

Supplementary Figure legends

Suppl. Figure 1. IRF1 is regulated in an NF-κB-dependent manner.

A172 glioblastoma transfected with control vector and IκBα-SR cells were treated for six hours (A) or indicated time points (B) with 5 μM BV6. Gene expression profiling was performed using Illumina Whole Genome Expression Beadchips Human HT12v4 and mean intensity of IRF1 mRNA signal of three independent experiments is shown (A). IRF1 mRNA was assessed by qRT-PCR and normalized to 28S rRNA expression (B). Data are presented as fold increase of untreated vector control cells. Mean + SD values of two independent experiments performed in duplicate are shown.

Suppl. Figure 2. siIRF1_1 does not affect IRF family members other than IRF1.

(A) Basal mRNA expression levels of IRF1, IRF2, IRF3, IRF5, IRF6 and IRF9 in MDA-MB-231, T24, Kym-1 and SK-N-AS cells were assessed by qRT-PCR and normalized to 28S rRNA expression. Data are presented as fold change of MDA-MB-231 cells. Mean + SD values of three independent experiments performed in duplicate are shown.

(B) Basal mRNA expression levels of IRF4, IRF7 and IRF8 in MDA-MB-231, T24, Kym-1 and SK-N-AS cells were assessed by qRT-PCR and normalized to 28S rRNA expression. Reh cells, untreated MV4-11 or IFNα-treated MV4-11 cells were used as positive controls. Data are presented as fold change of positive controls. Mean + SD values of two independent experiments performed in duplicate are shown.

(C) MDA-MB-231, T24, Kym-1 and SK-N-AS cells were transiently transfected with 5 nM siRNA against IRF1 or control siRNA. IRF1, IRF2, IRF3, IRF5, IRF6 and IRF9 mRNA levels were assessed by qRT-PCR and normalized to 28S rRNA expression. Data are presented as fold change of expression levels in MDA-MB-231 cells. Mean

+ SD values of three independent experiments performed in duplicate are shown. *P< 0.05; **P< 0.01, n.s. not significant.

Suppl. Figure 3. IRF1 expression is necessary for BV6-induced cell death.

MDA-MB-231 cells were transiently transfected with 5 nM siRNA against IRF1 or control siRNA. IRF1 mRNA levels were assessed by qRT-PCR and normalized to 28S rRNA expression (A). Data are presented as fold increase of untreated controls. Mean + SD of two independent experiments performed in duplicate are shown. Cell viability after treatment with BV6 at indicated concentrations for 72 hours was measured by MTT and is expressed as percentage of untreated controls (B). Apoptosis after treatment with BV6 at indicated concentrations for 72 hours was determined by DNA fragmentation of PI-stained nuclei using flow cytometry and the percentage of DNA fragmentation is shown (C). Mean + SEM of three to four independent experiments performed in duplicate are shown. *P< 0.05; **P< 0.01.

Suppl. Figure 4. Representative FACS profiles for DNA fragmentation and Annexin V/PI staining.

MDA-MB-231, T24, Kym-1 and SK-N-AS cells were transiently transfected with 5 nM siRNA against IRF1 or control siRNA. Cells were treated with 50 nM ((A) MDA-MB-231)), 25 nM ((B) MDA-MB-231), 4 μM (T24), 50 nM (Kym-1) or 200 nM (SK-N-AS) BV6 for 72 hours (A) or 24 hours (B). Apoptosis was determined by DNA fragmentation of PI-stained nuclei (A) or Annexin V/PI staining (B) using flow cytometry and representative FACS profiles are shown.

Suppl. Figure 5. IRF1 expression is necessary for BV6-induced apoptosis.

MDA-MB-231, T24, Kym-1 and SK-N-AS cells were transiently transfected with 5 nM siRNA against IRF1 or control siRNA. Cells were treated with 25 nM (MDA-MB-231), 4 μM (T24), 50 nM (Kym-1) or 200 nM (SK-N-AS) BV6 for 24 hours. Cell death was determined by Annexin-V/PI double staining and flow cytometric analysis. The percentage of Annexin V and/or PI-positive cells is shown with mean and SEM of three to four experiments performed in triplicate.

Suppl. Figure 6. BV6 induces TNFα mRNA and protein expression.

(A) MDA-MB-231 and T24 cells were treated with 50 nM BV6 (MDA-MB-231) or 4 μM (T24) BV6 for indicated time points. TNFα mRNA levels were assessed by qRT-PCR and normalized to 28S rRNA expression. Data are presented as fold increase of untreated controls. Mean + SEM of two independent experiments performed in duplicate are shown.

(B) MDA-MB-231 and T24 cells were treated with 50 nM BV6 (MDA-MB-231) or 4 μM (T24) BV6 for 15 hours. Cell culture supernatants of treated cells were harvested and assessed for TNFα proteins levels by ELISA. Data are presented as fold increase of untreated controls. Mean + SEM of two to three independent experiments performed in duplicate are shown.

Suppl. Figure 7. BV6-induced cell death depends on TNFR1.

T24 cells were transiently transfected with 5 nM siRNA against TNFR1 or control siRNA. Protein levels of TNFR1 were analyzed after 24 hours by Western blotting, GAPDH served as loading control (A). Apoptosis after treatment with BV6 at indicated concentrations for 72 hours was determined by DNA fragmentation of PI-stained nuclei using flow cytometry and the percentage of DNA fragmentation is

shown (B). Mean + SD of three to four independent experiments performed in duplicate are shown. *P< 0.05; **P< 0.01.

Suppl. Figure 8. IRF1 is required for BV6-mediated TNFα induction.

T24 cells were transiently transfected with 5 nM siRNA against IRF1 or control siRNA.

(A) Protein levels of IRF1 were analyzed after 24 hours by Western blotting. β-Actin served as loading control.

(B) Cells were treated with 4 μM BV6 for indicated time points. TNFα mRNA levels were assessed by qRT-PCR and normalized to 28S rRNA expression (B). Data are presented as fold increase of untreated control siRNA cells. Mean + SEM of four independent experiments performed in duplicate are shown.

(C) Cells were treated with 4 μM BV6 for 15 hours, cell culture supernatants were harvested and assessed for TNFα proteins levels by ELISA. Data are presented as fold change of treated control siRNA cells. Mean + SD of four independent experiments performed in duplicate are shown.

(D) Cells were treated with 4 μM BV6 for 15 hours. 50 pg/ml TNFα were additionally added after 15 hours and cells were analyzed after 48 hours in total. Apoptosis was determined by DNA fragmentation of PI-stained nuclei using flow cytometry and the percentage of DNA fragmentation is shown with mean + SD of three independent experiments performed in duplicate. *P< 0.05; **P< 0.01.

Suppl. Figure 9. IRF1 selectively controls BV6-induced NF-κB target genes.

T24 cells were transiently transfected with 5 nM siRNA against IRF1 or control siRNA and treated for indicated time points with 4 μM BV6. IL-8, p100 and RelB mRNA

levels were assessed by qRT-PCR and normalized to 28S rRNA expression. Data are presented as fold increase of untreated control siRNA cells. Mean + SEM of four independent experiments performed in duplicate are shown.

Suppl. Figure 10. Knockdown of IRF1 and p65.

MDA-MB-231 and Kym1 cells were transiently transfected with 5 nM siRNA against IRF1, p65 or control siRNA and treated with 50 nM (MDA-MB-231) or 100 nM (Kym1) BV6 for 12 hours. IRF1 (A) and p65 (B) mRNA levels were assessed by qRT-PCR and normalized to 28S rRNA expression. Mean + SD values of two independent experiments performed in duplicate are shown.

Suppl. Figure 11. IRF1 and p65 cooperatively regulate BV6-induced TNFα upregulation and NF-κB reporter activity.

(A) MDA-MB-231 cells were transiently transfected with 5 nM siRNA against IRF1, p65 or control siRNA and treated with 50 nM BV6 for 12 hours. TNFα mRNA levels were assessed by qRT-PCR and normalized to 28S rRNA expression. Data are presented as fold increase of untreated control siRNA cells. Mean + SD values of two independent experiments performed in duplicate are shown.

(B) MDA-MB-231 cells stably expressing a GFP-labeled NF-κB reporter construct were transiently transfected with 5 nM siRNA against IRF1, p65 or control siRNA and treated for 15 hours with 50 nM BV6. NF-κB reporter activity was assessed by flow cytometry and is displayed as fold increase of untreated control siRNA cells. Mean + SD values of three independent experiments performed in duplicate are shown. $^{*}P < 0.05$; $^{**}P < 0.01$.

Suppl. Figure 12. p65 is the main NF-κB subunit involved in BV6-induced cell death.

MDA-MB-231 cells were transiently transfected with 5 nM siRNA against p65, RelB, c-Rel or control siRNA. p65, RelB and c-Rel mRNA levels were assessed by qRT-PCR and normalized to 28S rRNA expression (A). Mean + SEM values of two independent experiments performed in duplicate are shown. Apoptosis after treatment with 50 nM BV6 for 72 hours was determined by DNA fragmentation of PI-stained nuclei using flow cytometry and the percentage of DNA fragmentation is shown (B). Mean + SD of three independent experiments performed in duplicate are shown. *P< 0.05; **P< 0.01.

Suppl. Figure 13. IRF5 is partially involved in BV6-mediated cell death and TNFα induction.

(A) MDA-MB-231 and T24 cells were transiently transfected with 5 nM siRNA against IRF5 or control siRNA. IRF5 mRNA levels were assessed by qRT-PCR and normalized to 28s rRNA expression. Data is presented as fold change of untreated controls. Mean + SD values of four to five experiments are shown.

(B-C) MDA-MB-231 and T24 cells were transiently transfected with 5 nM siRNA against IRF5 or control siRNA and treated with 20 nM (MDA-MB-231) or 1 µM (T24) BV6. Apoptosis was determined by DNA fragmentation of PI-stained nuclei using flow cytometry and the percentage of apoptosis is shown (B). Mean + SD values of three to four experiments are shown. TNFα mRNA levels were assessed by qRT-PCR and normalized to 28s rRNA expression. Data is presented as fold change of treated control siRNA cells (C). Mean + SD values of four to five experiments are shown.*P< 0.05; **P< 0.01.

(D) MDA-MB-231 and T24 cells were treated with 50 nM (MDA-MB-231) or 4 μM (T24) BV6 for indicated time points. IRF5 mRNA levels were assessed by qRT-PCR and normalized to 28S rRNA expression. Data are presented as fold increase of untreated controls. Mean + SD of two independent experiments are shown.

Suppl. Figure 14. IRF1 gene copy number in MDA-MB-231 and T24 cells.

Genomic DNA of HEK293T, MDA-MB-231 and T24 cell was isolated. IRF1 gene copy number was assessed by qRT-PCR and normalized to RNAseP. Mean + SD values of two independent experiments performed in duplicate are shown.

Suppl. Figure 15. BV6 induces slight nuclear accumulation of IRF1.

(A) MDA-MB-231 T24 and SK-N-AS cells were treated with 50 nM (MDA-MB-231), 4 μM (T24) or 200 nM (SK-N-AS) BV6 for indicated time points. IRF1 mRNA levels were assessed by qRT-PCR and normalized to 28S rRNA expression. Data are presented as fold increase of untreated controls. Mean + SD of two to three independent experiments performed in duplicate are shown.*$P < 0.05$.

(B) MDA-MB-231 and T24 cells were treated with 50 nM (MDA-MB-231) or 4 μM (T24) BV6 for two hours. Medium containing 25 μg/ml CHX was applied and samples were harvested at indicated time points. IRF1 expression was analyzed by Western blotting. β-Actin was used as loading control. Asterisks indicate unspecific bands.

(C) MDA-MB-231 and T24 cells were treated with 50 nM (MDA-MB-231) or 4 μM (T24) BV6 for indicated time points. IRF1 expression levels were analyzed in the nucleus or cytosol by Western blotting. α-Tubulin served as purity control for cytoplasmic fractions, lamin A/C for nuclear fractions and β-Actin as loading control for both fractions. Asterisk indicates unspecific bands.

(D) Quantification of nuclear IRF1 levels. Densitometric analysis of Western Blots shown in panel (C). Nuclear IRF1 levels were normalized to β-Actin. Mean + SD of two independent experiments are shown.

Supplementary methods

Copy number assay

IRF1 gene copy number was determined using TaqMan Copy Number Assay (Life technologies, Hs05974060_cn) according to the manufacturer's instructions. The data were normalized on TaqMan Copy Number Reference Assay, RNaseP (#4403326). Genomic DNA was extracted using Pure Link DNA Mini Kit (#K182001) from Life technologies.

Gene expression profiling

Gene expression profiling was performed as previously described [26]. A172 control vector and IκBα-SR cells were treated for 6 hours with 5 μM BV6. Total RNA was extracted using peqGOLD Total RNA kit from Peqlab Biotechnologie GmbH (Erlangen, Germany) according to the manufacturer's instructions, including DNase digestion. The experiment was independently performed three times. Gene expression profiling was performed using Illumina Whole Genome Expression Beadchips Human HT12v4. For target gene identification, genes were ranked according their fold upregulation in vector control cells. Genes that were similarly regulated in IκBα-SR cells or didn't show significant differences in expression levels in both treated conditions were removed from the data set ($p < 1.25x\ 10^{-6}$). Promoter regions of validated genes were analyzed for NF-κB binding sites and Gene Set enrichment analysis verified top hits to be NF-κB target genes. Genes were validated via qRT-PCR and/or immunoblotting.

Determination of apoptosis by Annexin-V/PI staining.

Annexin-V/PI staining was performed according to the manufacturer's instructions (BD Biosciences) and analyzed by flow cytometry.

siRNA-mediated gene silencing.

For knockdown experiments the following Silencer Select siRNAs (Life Technologies) were used: s11918, s11919 for RelB, s11905, s11907 for c-Rel, s7513, s7515, for IRF5).

Densitometry.

IRF1 protein expression was quantified by densitometry using Image J digital imaging software.
